# Transcriptomic and Physiological Analyses Reveal Potential Genes Involved in Photoperiod-Regulated β-Carotene Accumulation Mechanisms in the Endocarp of Cucumber (*Cucumis sativus* L.) Fruit

**DOI:** 10.3390/ijms232012650

**Published:** 2022-10-21

**Authors:** Hesbon Ochieng Obel, Chunyan Cheng, Zhen Tian, Martin Kagiki Njogu, Ji Li, Shengli Du, Qunfeng Lou, Junguo Zhou, Xiaqing Yu, Joshua Otieno Ogweno, Jinfeng Chen

**Affiliations:** 1 State Key Laboratory of Crop Genetics and Germplasm Enhancement, College of Horticulture, Nanjing Agricultural University, Nanjing 210095, China; 2 China-Kenya Belt and Road Joint Laboratory on Crop Molecular Biology, Nanjing Agricultural University, Nanjing 210095, China; 3 Department of Plant Science, Chuka University, Chuka 60400, Kenya; 4 State Key Laboratory of Vegetable Germplasm Innovation, Tianjin Kernel Cucumber Research Institute, Tianjin 300192, China; 5 College of Horticulture and Landscape, Henan Institute of Science and Technology, Xinxiang 453003, China; 6 Department of Crops, Horticulture and Soil Science, Egerton University, Njoro 20115, Kenya

**Keywords:** Xishuangbanna cucumber, transcriptomics, photoperiod, β-carotene, WGCNA, light signaling

## Abstract

The accumulation of carotenoids in plants is a key nutritional quality in many horticultural crops. Although the structural genes encoding the biosynthetic enzymes are well-characterized, little is known regarding photoperiod-mediated carotenoid accumulation in the fruits of some horticultural crops. Herein, we performed physiological and transcriptomic analyses using two cucumber genotypes, SWCC8 (XIS-orange-fleshed and photoperiod-sensitive) and CC3 (white-fleshed and photoperiod-non-sensitive), established under two photoperiod conditions (8L/16D vs. 12L/12D) at four fruit developmental stages. Day-neutral treatments significantly increased fruit β-carotene content by 42.1% compared to short day (SD) treatments in SWCC8 at 40 DAP with no significant changes in CC3. Day-neutral condition elevated sugar levels of fruits compared to short-day treatments. According to GO and KEGG analyses, the predominantly expressed genes were related to photosynthesis, carotenoid biosynthesis, plant hormone signaling, circadian rhythms, and carbohydrates. Consistent with β-carotene accumulation in SWCC8, the day-neutral condition elevated the expression of key carotenoid biosynthesis genes such as *PSY1*, *PDS*, *ZDS1*, *LYCB*, and *CHYB1* during later stages between 30 to 40 days of fruit development. Compared to SWCC8, CC3 showed an expression of DEGs related to carotenoid cleavage and oxidative stresses, signifying reduced β-carotene levels in CC3 cucumber. Further, a WGCNA analysis revealed co-expression between carbohydrate-related genes (pentose-phosphatase synthase, β-glucosidase, and trehalose-6-phosphatase), photoperiod-signaling genes (*LHY*, *APRR7/5*, *FKF1*, *PIF3*, *COP1*, *GIGANTEA*, and *CK2*) and carotenoid-biosynthetic genes, thus suggesting that a cross-talk mechanism between carbohydrates and light-related genes induces β-carotene accumulation. The results highlighted herein provide a framework for future gene functional analyses and molecular breeding towards enhanced carotenoid accumulation in edible plant organs.

## 1. Introduction

Carotenoids are indisputably one of the vital plant pigments that contribute to human nutrition and health by providing dietary provitamin A. Vitamin A improves vision and provides an antioxidant effect against cardiovascular and cancer disorders [1,2]. Previous studies have described carotenoid biosynthesis and regulation as well as its stimulation by environmental factors such as light, temperature, drought, salt stress, hormones, photoperiods, developmental cues, and post-transcriptional feedback regulations in plants [3,4,5]. Plants have developed mechanisms to perceive light signals, such as light intensity, variable wavelengths, direction, and the circadian clock. The effect of photomorphogenic genes on carotenoid biosynthesis involves complex interactions [5,6]. In a nutshell, a circadian rhythm consists of interlocking transcription or translation loops that regulate photomorphogenic genes comprising phytochromes (PHY) and phytochrome-interacting factors (PIF), cryptochromes (CRY), phototropin, and UVR8 signals. Plant photoreceptors receive circadian signals during plant etiolation and subsequently construct a passage of intracellular second messenger processes by inducing signals to control carotenoid biosynthesis [7].

Carotenoid accumulation is coordinated by photoreceptor signaling via controlling phytoene synthase 1 (*PSY1*), a rate-limiting step in carotenoid biosynthesis [8]. A ring-finger ubiquitin E3 ligase called constitutive photomorphogenic 1 (*COP*1) plays a key role in photomorphogenesis. *COP1* connected to the suppressor of *PHYA* (*SPA*) and other COP1 co-factors is inhibited by the photoreceptor in order to limit the generation of photomorphogenesis. *PIF1* and other transcription factors from the *PIF* family lower the accumulation of carotenoids by suppressing the gene encoding phytoene synthase in the dark [9]. In the tomato plant, the downregulation of *COP1-LIKE* resulted in a greater carotene accumulation whereas suppressing *HY5* expression decreased the quantities of carotenoids [10]. Light-induced isomerization caused the formation of lycopene in orange-headed Chinese cabbage exposed to light, implying that light can replace carotenoid Isomerase (*CRTISO)* activity [11]. The *PIF1/HY5* regulatory module is involved in carotenoid production photomorphogenesis in *Arabidopsis thaliana* (L.) Heynh and the fruit ripening of tomatoes. It is currently unknown whether the *PIF1/HY5* function that appears at the start of tomatoes’ ripening is an ancestral trait shared by all fleshy fruits or if it was specifically identified from the photomorphogenesis network in tomatoes, as well as whether similar responses can be extrapolated to other crop species. Other light-responsive genes, pseudo-response regulators (*PRR*), have been reported to be involved in plant growth and development, responses to environmental stress, and metabolite biosynthesis. As an essential component of the circadian clock, the *PRR* gene family plays a critical role in the plant photoperiod pathway. Promoter cis-element analysis of maize *PRR* genes demonstrated that they might be involved in multiple signaling transduction pathways, such as light, abiotic stress, and hormone regulations in plants [12]. In another genome-wide study, an analysis of a light-related gene, the ring-H2 finger E3 ligase in cotton (*Gossypium hirsutum*), revealed its role in fiber development and phytohormone and abiotic stress responses [13]. The circadian clock can create an oscillator’s action to deal with clock time; this may also create a system where sugars affect biological processes, which are thought of as the outputs of biological oscillators. All of these activities are due to the fact that sugars have the ability to directly control the circadian clock’s outputs, despite having no function in the circadian rhythm.. Sugars in the long-term response pathway have been found to alter the circadian clock associated 1 (*CCA1*), strengthening the rhythmicity in *A*rabodopsis leaves during the night and activating the activity of Gigantea (*GI*) [14]. CCA1 protein respond to environmental light signals and may, therefore, maintain the circadian rhythms and give plants the ability to acclimatize to the environment [15,16].

Perceiving incoming environmental information is critical for optimizing plant growth and development. Numerous B-box proteins (BBXs) are crucial for light-dependent plant developmental processes. For instance, the essential component of photomorphogenesis, *HY5*, interacts with *AtBBX21* [17], which also binds to the T/G-box cis-element in the promoter of *HY5* to activate transcription [18,19]. Interestingly, while *HY5* can bind to the promoter area of *BBX11*, *BBX11* can also positively influence the expression of *HY5* by directly binding to its promoter region. At the transcriptional level, the proteins BBX11*, BBX21*, and *HY5* collaborate to positively regulate photomorphogenesis [18]. In order to control the biosynthesis of anthocyanins and other plant metabolites such as carotenoids and to take part in signaling cascades, BBX proteins are therefore anticipated to play important functions. The Kelch repeat F-box (*KFB*) proteins are also essential for controlling a variety of physiological and biochemical processes in plants, such as growth and development, stress responses, and secondary metabolism. Several *StKFB* genes were found to express differently in potato tubers with yellow-, red-, and purple-fleshed cultivars, respectively. This suggests that these genes may be crucial in the control of anthocyanin production in potatoes [20] and may also participate in the carotenoid pathway. Although light signals regulate the content of sugars and a variety of other fruit constituents such as anthocyanins and carotenoids in both leaf and fruit tissues [21,22], little is known about the effect of photoperiod shifts on the molecular regulation of metabolites such as carotenoids in cucumbers.

The Xishuangbanna cucumber (*Cucumis sativus* L. var. Xishuangbannesis Qi et Yuan; XIS, hereinafter) is a typical photoperiod-sensitive botanical variety that requires short days for the induction of female flowers [23,24]. Most importantly, mature XIS fruit (≥40 days after flowering) develop an orange-fleshed endocarp with a significant accumulation of β-carotene content (700 μg/100 g, fresh weight) compared to commercial varieties (20–30 μg/100 g, fresh weight) [25]. While the genetic factor conferring β-carotene accumulation in XIS cucumbers has been reported [26,27], the impacts of the photoperiod on the carotenoid accumulation in XIS remains elusive. The objective of this study was to unravel the physiological and molecular mechanisms underlying photoperiod-regulated β-carotene accumulation in the endocarp of XIS cucumbers.

In this study, we conducted experiments in a controlled environment where the impact of independent and physical components such as the photoperiod can only be evaluated in a manner that excludes interaction with other environmental factors. RNA sequencing (RNA-seq) was conducted to analyze the transcripts and related genes in the photoperiod-sensitive, orange-fleshed cultivar (SWCC8; XIS type) and the photoperiod-insensitive, white-fleshed cucumber (CC3) during four fruit developmental stages. DEGs associated with carbohydrate transport and metabolism, and light signaling, were expressed during the fruit maturity stages of the XIS cucumber (SWCC8), depicting the degradation of carotenoids. DEGs associated with oxidative stresses were highly expressed in CC3. A co-expression analysis using WGCNA further revealed the genes involved in photoperiod-mediated carotenoid biosynthesis. Further, the validation of RNA-Seq data of carotenoid-related genes and selected photomorphogenic genes using qRT-PCR confirmed the validity of our transcriptome data. Herein, we show that the photoperiod constitutes multiple pathways to regulate β-carotene accumulation in XIS cucumbers in comparison to the non-orange-fleshed and photoperiod-insensitive cultivar (CC3). Therefore, we schematically postulated the concept of photoperiod-induced β-carotene accumulation in the fruit endocarp of XIS cucumbers. Insights into the mechanisms that regulate carotenoid biosynthesis in response to changes in photoperiod are fundamental and constitute the first steps towards novel candidate gene identification and the molecular breeding of quality fruits.

## 2. Results

### 2.1. Photoperiod Affects Flesh Color Changes and Accumulation of β-Carotene (μg/g, fw) during Fruit Development

A differential progressive flesh color change of SWCC8 was observed under the photoperiod treatment while the fruits of CC3 cultivar did not reveal any noticeable flesh color changes throughout the fruit developmental period (Figure S1). Under day-neutral conditions, the cultivar SWCC8 manifested an early color change at 20 DAP after pollination with an orange color progressively becoming more intense and peaking at 40 DAP. The change to an orange flesh color for SWCC8 under short-day (SD) conditions became noticeable at 30 DAP with a mild orange color intensity compared to day-neutral conditions after 40 DAP.

**Figure 1:**
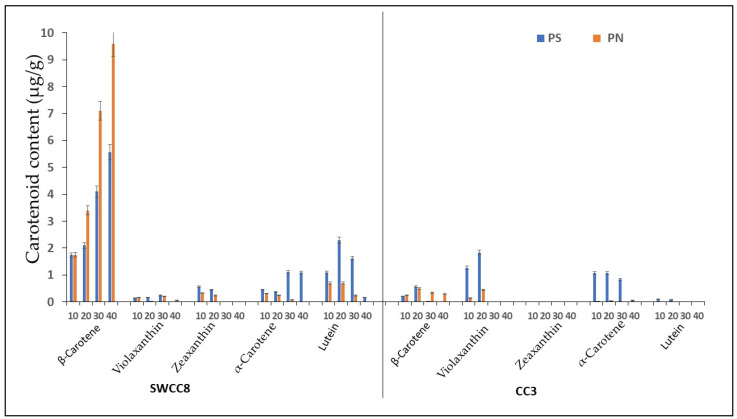
Carotenoid content (μg/g) and composition in the endocarp of WCC8 and CC3 under photoperiodic regimes. Data are means ± sd from analysis of carotenoid content in the endocarp of WCC8 and CC3 under two photoperiods. Vertical error bars represent the standard error of means (*n* = 3); 10, 20, 30, and 40 are days after pollination; PS—short day photoperiod; PN—Neutral day photoperiod.

A carotenoid analysis showed that β-carotene was the dominant carotenoid in the XIS cucumber (Figure 1). An analysis of the β-carotene content corresponded with the observed flesh color phenotypes under the two growing conditions. The day-neutral treatment increased orange pigmentations in SWCC8 with high β-carotene concentrations compared to short days reaching 9 μg/g and 6 μg/g fw at 40 days after anthesis (DAA), while CC3 had a significantly lower (0.03–0.57 μg/g FW) β-carotene content. Therefore, the production of SWCC8 under differential photoperiod regimes significantly influenced the concentration of β-carotene with no significant difference recorded in CC3 under the two photoperiod conditions. Other carotenoid pigments, such as violaxanthin, zeaxanthin, α-carotene, and lutein, were observed under different developmental stages but at lower concentrations.

### 2.2. Photoperiod Treatments Modulate Carbohydrate Levels in the Endocarp of Cucumbers

**Figure 2:**
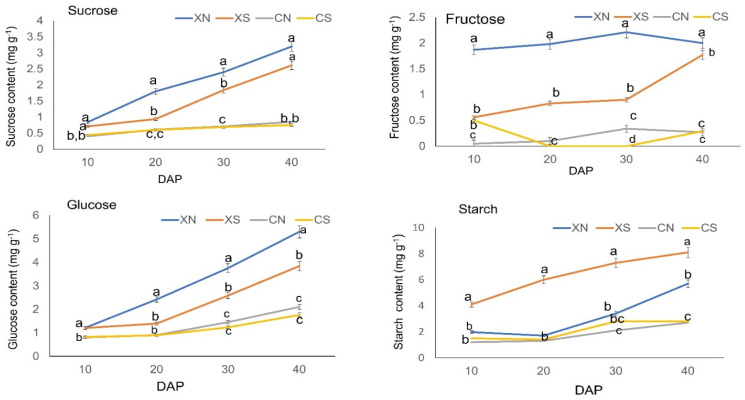
Carbohydrate profiles. The relative content of sucrose, fructose, glucose, and starch is influenced by photoperiod levels in the endocarp of the two cucumber varieties over time (DAP; days after pollination). Vertical bars represent the standard error of means (*n* = 3). Means with the same letters within each sampling day are not significantly different according to Tukey’s *t*-test (*p* ≤ 0.05). XN-SWCC8 under day neutral; XS—SWCC8 under a short day; CN—CC3 under day neutral; CS—CC3 under short day.

Since assimilates are important for fruit metabolism, we expected that carbohydrates might be involved in photoperiod changes. The contents of sucrose, fructose, and glucose were significantly higher in XN than XS after 20 DAP throughout the fruit development stages (Figure 2). The sucrose, fructose, and glucose levels were significantly higher in SWCC8 than in CC3 after 10 DAP. CC3 did not show a significant difference in sugar levels between the short-day (CS) and day-neutral (CN) treatments possibly due to its insensitivity to the photoperiod. On the other hand, starch was significantly higher under the short-day (XS) than day-neutral treatment (CN) in SWCC8, and a similar trend was observed in C33 although with no significant difference. Starch accumulation remained as reservation storage of carbohydrates under the short-day treatment and remobilized under the day-neutral treatment.

The findings demonstrate that a portion of the cucumber endocarp’s sugar metabolism is subject to photoperiodic regulation and becomes endogenously structured over time in SWCC8, but they are only marginally altered in CC3. The results also show a varietal response to the photoperiod regarding carbohydrate metabolism.

### 2.3. Overview of Cucumber Transcriptomic Sequencing in Responses to Photoperiod Changes

Transcriptome sequencing of XIS (SWCC8) and CC3 comprised four developmental stages, and two photoperiod conditions with three biological replicates were used to construct 16 libraries. The transcriptome sequencing of the 48 samples yielded 206.14 billion high-quality clean reads ranging from 38.6 to 46.9 million per sample and was mapped onto the cucumber genome database (http://cucurbitgenomics.org/organism/20, accessed on 10 July 2021). Approximately 96.17% of the clean reads were mapped to the reference cucumber genome with more than 94 uniquely mapped. A total of 309.22 Gb of clean data was obtained with 93.62% of bases scoring Q30 and above, thereby meeting the criterion for gene discovery (Datasheet S1). To confirm the accuracy of the expression data, the Pearson correlation coefficient was computed after the gene expression levels were standardized by FPKM. This study revealed that the correlation coefficient in the gene expression levels from three biological replicates of each line was greater than 0.84 and that all the R2 values between the biological replicates were greater than those outside of the biological replicates (Figure S2). According to the principal component analysis (PCA), biological replications have a propensity to cluster together (Figure S3). The correlation coefficients and PCA indicated that the expression patterns of the repeated samples are similar.

### 2.4. Differential Gene Expression during Photoperiod Induction

A total of 54,578 unique DEGs were identified in SWCC8 under the short-day treatment (XS) with 27,430 up-regulated and 27,148 down-regulated (Table S1). On the other hand, SWCC8 under the day-neutral treatment (XN) yielded a comparatively lower number of DEGs than XS, comprising a total of 12,959 DEGs, with 5152 up-regulated and 7807 down-regulated (Table S1). However, a similar trend was observed for CC3 under the same conditions, with a lower number of DEGs compared to SWCC8. Under the short-day treatment, CC3 (CS) resulted in a total of 17,010 DEGs with 8880 up-regulated while 8130 were down-regulated. Under the neutral-day treatment, CC3 (CN) had a total of 15,961 DEGs, comprising 7064 up-regulated and 8897 down-regulated DEGs (Table S1). Interestingly, in both genotypes, more up-regulated genes were observed under the short-day than neutral-day treatments, while the down-regulated genes depicted an opposite trend.

### 2.5. Gene Expression Trend Analysis, GO, and KEGG Classifications

**Figure 3:**
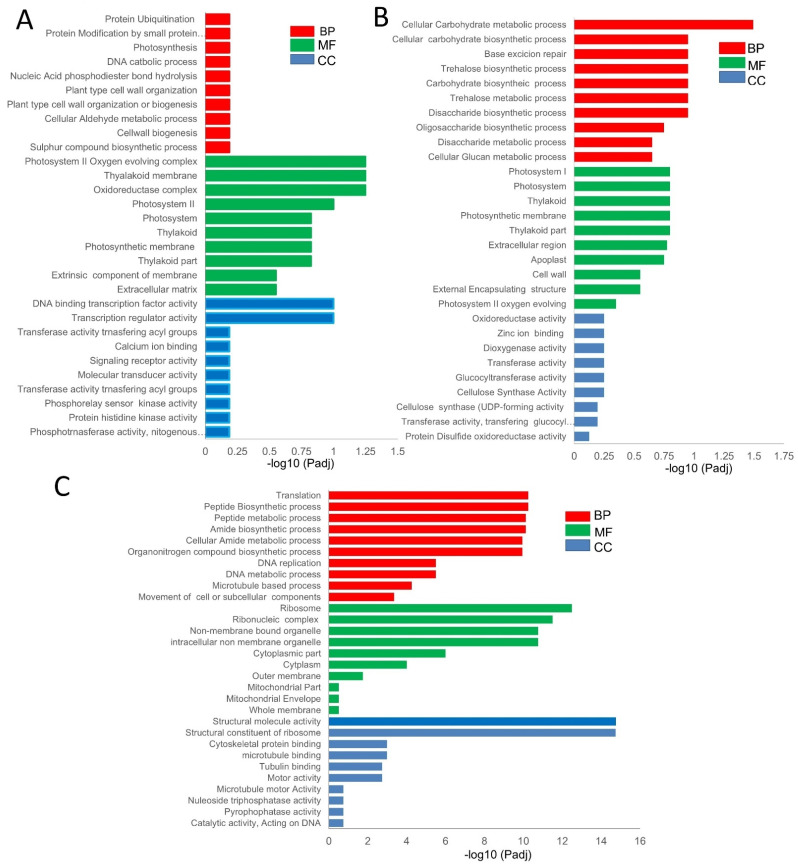
Gene ontology (GO) enrichment of differentially expressed genes in timepoint pairwise comparisons. (**A**) Shared GO in the pairwise comparisons between XS_vs_XN of 160 DEGs. (**B**) Common shared GO among timepoint pairwise comparisons of 30 DEGs within XN. (**C**) Common shared GO among timepoint pairwise comparisons of 1351 DEGs within XS. Red, green, and blue bars represent biological processes, molecular functions, and cellular components. The *y*-axis represents different GO terms and log10 (adj) in the x-axis.

To better understand the function of the DEGs in the pairwise comparisons, we performed a gene ontology (GO) enrichment analysis and classified them under molecular function, biological activity, and cellular component. Comparing the XS-vs-XN, the top significantly enriched GO sub-categories at all time point comparisons in the biological process were impacted with respect to “protein ubiquitination”, “photosynthesis”, “DNA catabolic process”, and “nucleic phosphodiester bond hydrolysis.” In the molecular function category, “DNA binding transcription factor activity”, “transferase activity “, and “calcium ion binding and signal receptor activity” were enriched (Figure 3A, Datasheet S2). Some GO terms, such as “photosystem I”, “photosynthesis”, and “chloroplast thylakoid”, related to light reaction were significantly enriched in the XS_20 vs. XN_20 and XS_40 vs. XN_40 (Datasheet S2). For XN, the GO within the biological processes was enriched in “carbohydrate metabolic processes”, thus constituting the larger portion of the DEGs while cellular components comprised “photosystem”, and “thylakoid and extracellular region”, and molecular functions were enriched in “transport activity” and “enzyme regular activity and ion binding” (Figure 3B, Datasheet S3). The DEGs common within XS among the time point-pairwise comparison were mainly involved in “translation”, “peptide biosynthesis”, “amide biosynthetic process”, and “organonitrogen compound biosynthetic process”, among others (Figure 3C, Datasheet S3). Additionally, genes linked to “carbon metabolism” and “starch and sucrose metabolism” were specifically enriched at XN 40 DAP vs. XN 30 DAP, demonstrating a significant differential in carbohydrate metabolism between XS and XN at this time. At all-time points, the number of genes encoding “photosynthesis-antenna proteins” was enriched. This pathway includes the circadian clock-controlled genes FLA-VIN-BINDING, KELCH REPEAT, and F-BOX 1 (FKF1), suggesting that circadian rhythm genes may have photoperiod-sensitive functions in the endocarp of cucumbers. Protein *PHYTOCHROME KINASE SUBSTRATE* 1 (*PKS1*) was induced in XIS under XN; it is a phototropin and its up-regulation suggests that the *PKS1* proteins may provide a molecular link in the endocarp of XIS cucumbers (Table S2).

For CS_vs_CN pairwise comparisons, the GO terms in the biological process were implicated with respect to “amine”, “cellulose”, “polysaccharide” “beta-glucan metabolic” and “biosynthetic process” (Figure S4A). The common GO terms that were either up- or down-regulated at all time points within CS include “amine metabolic process”, “response to biotic stimulus”, and the response to stress, among others in the biological process (Figure S4B). The CN Go terms in the metabolic process category were enriched in the “organophosphate biosynthetic process”, “alcohol metabolic process”, and the “organic hydroxy compound metabolic” process among others. (Figure S4C). Next, we compared the GO terms between genotypes under similar photoperiod conditions, that is, XS_vs_CS and XN_vs_CN, to pinpoint those common to the two genotypes and under the same photoperiodic responses. Common GO terms within the XS_vs_CS comparison were enriched in the “cellulose metabolic process”, “polysaccharide biosynthetic process”, and “β-glucan metabolic process” among others, implying that they may represent the ‘core transcriptome’ associated with major differences between the two cucumber cultivars (Figure S5A). On the other hand, the GO terms commonly expressed within the XN_vs_CN comparison are related to proteolysis, transport, tRNA processing, and ribosome biogenesis (Figure S5B).

**Figure 4:**
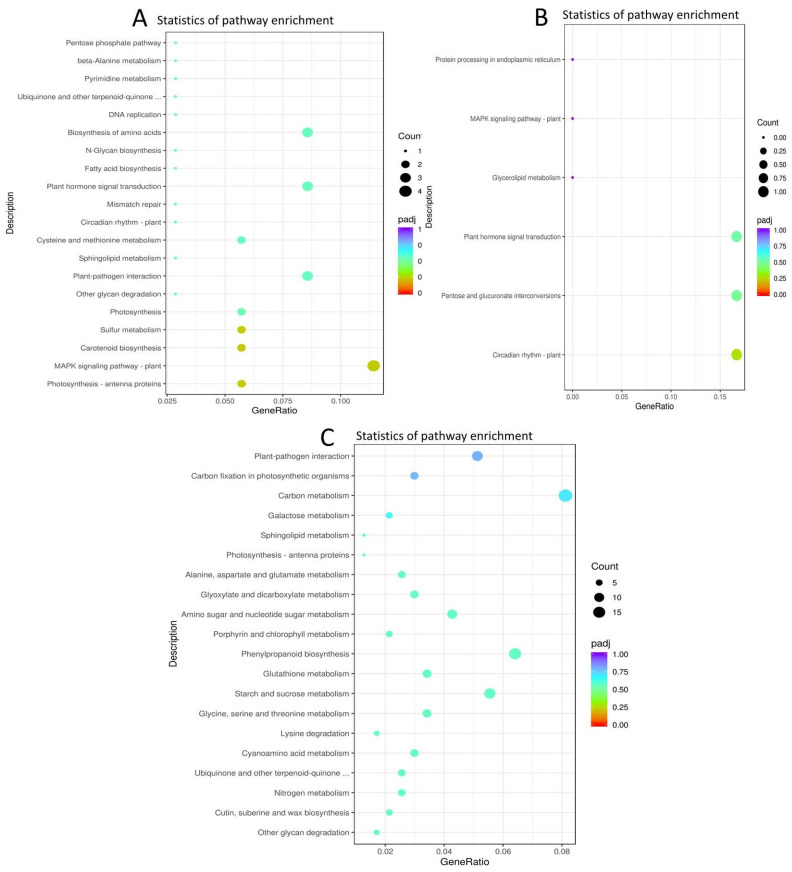
Significantly enriched KEGG pathways of DEGs in timepoint pairwise comparisons. (**A**) KEGG pathways of genes are commonly shared within timepoint pair-wise comparisons of XS_vs_XN. (**B**) KEGG pathways enriched in XN_vs_CN time point pairwise comparisons. (**C**) KEGG pathways enriched in XS_vs_CS pairwise comparisons.

We conducted a KEGG (Kyoto Encyclopedia of Genes and Genomes) analysis, and the result showed that genes encoding “photosynthesis-antenna”, “MAPK signaling”, “carotenoid biosynthesis”, and “Sulphur metabolism and photosynthesis proteins, circadian rhythm, and plant hormone signal transduction” were commonly enriched within the timepoint pairwise comparison of XS_vs_XN (Figure 4A, Datasheet S4). Considering the commonly enriched pathways in each photoperiod condition among the time points pairwise comparisons, KEGG pathways related to the ribosome, DNA replication, glyoxylate, decarboxylate, and carotenoid biosynthesis were commonly enriched within XS (Figure S6A) while, “starch and sucrose metabolism”, “sesquiterpenoid and triterpenoid biosynthesis” and “base excision repair” were commonly enriched within XN at all timepoint pairwise comparisons (Figure S6B).

The shared DEGs between the pairwise comparisons between XN_vs_CN showed that the enriched KEGG pathways were related to “circadian rhythm-plant”, “pentose and glucose” interconversions, “plant hormone signal transduction”, and “glycerolipid metabolism and MAPK signaling pathway-plant “(Figure 4B, Datasheet S5). On the other hand, the shared DEGs in XS_vs_CS were enriched in the KEGG pathways related to “cutin, suberin, “wax biosynthesis”, “nitrogen metabolism”, “ubiquinone and other terpenoid quinones”, “starch and sucrose metabolism”, and “photosynthesis antennae proteins”, among others (Figure 4C, Datasheet S5).

### 2.6. DEGs Involved in Light-Signaling Perceptions and Circadian Rhythm

Photoreceptors belonging to several groups, such as phytochromes (*PHYs*), crypto-chromes (*Crys*), phototropin (PHOTs), and UV-resistance loci, have been found to react to light signals. We found differential expression levels of protein Phytochrome kinase substrate 1,4-like *(PKS1* and *PKS4*), Phytochrome (*PHYB* and *PHYC*), *CRY*, and protein kinase family protein (*PKS1*) (*CRY* showed up-regulation under XN and down-regulation under XS at all time points, while it shows fluctuating patterns in CC3, and was up-regulated at CS_40 and CN_30. *PHY* unigenes tended to depict opposite expression patterns relative to CRY (Figure S7).

A network analysis of DEGs in the circadian rhythm showed a significant up-regulation of Arabidopsis Pseudo-response regulator (A*PRR5*) and Late Elongated Hypocotyl (*LHY*) and a down-regulation of GIGANTEA (*GI*) and Chalcone synthase (*CHS*). Circadian rhythm genes comprising *APRR7*, *APRR5*, *GIGANTEA1,2*, and Flavin-binding Kelch repeat, F-box 1 (*FKF1*) showed higher expression in the XS over XN endocarps but with fluctuations patterns at different time points (Figure S7; Datasheet S6). For example, *FKF1* showed higher expression at XS_30 DAP and XS_40 DAP. *LHY-like*, flowering locus -Time (*FL-T*), and Early flowering-3 (*EF3*) showed higher expression in XN than in XS, with an undulating pattern along time points. The expression levels of *COP1_like* were generally low in either treatment and genotype but it tended to have a high expression at the early stages of fruit growth (10 DAP) for both XS and XN. Elongated hypocotyl (*HY5*) showed up-regulation at XS_10 DAP and downregulation in the subsequent stages of fruit development, while its expression was always higher under XN. The *HY*5 unigene (CsaV3. 2G031960) constantly showed higher expression levels in the CC3 cultivar than in XIS. Other circadian clock genes such as E3-*COP1_Like*, *TCP21-like*, and *CP7-like* were more highly expressed in the CC3 cultivar than in XIS, with *E3-COP1* showing a higher expression in CS, while *TCP21* and *TCP7-like* show higher expressions in CN (Figure S7). The presence of these important circadian genes in our DEGs suggests that they might play a critical role in cucumber endocarp development and carotenogenesis under a photoperiodic responses.

### 2.7. DEGs Involved in Carbohydrate Metabolism Induced by Photoperiod

**Figure 5:**
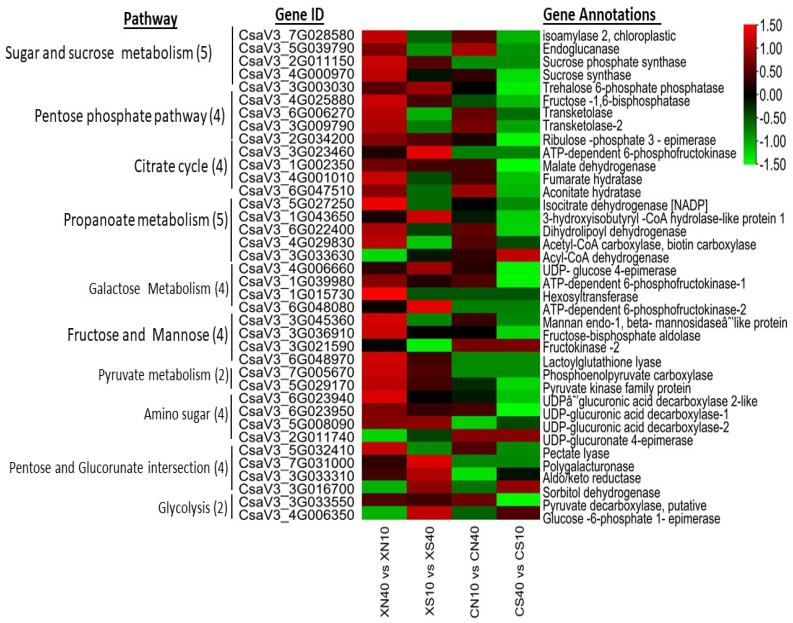
Heatmap of selected top DEGs of carbohydrate-related genes in timepoint pairwise comparisons between 10 vs. 40 DAP within photoperiod condition and genotype. The numbers in the parentheses in each pathway are the number of top DEG in each carbohydrate pathway.

The genes involved in carbohydrate metabolism were dominantly expressed in the endocarp at all times under the two photoperiod conditions and corroborated the measured sugar levels. We classified the major carbohydrate into 11 classes (Figure S8A). Starch and sucrose-related genes were the dominant categories comprising 92 DEGs. Therefore, we further analyzed the starch and sucrose-related genes and found that the β-glucosidase (18), trehalose-6-phosphatase (11), endoglucanase (10), glucan endo1,3-β-glucosidase (5), 1,4, α-glucan branching (4), and 1,4, α glucan phosphorylase (4) categories were the major gene categories with differential expression patterns (Figure S8B). Trehalose-6-phosphatase (T6P) gene plays a significant role in light signaling by suppressing the expression of Phytochrome Interacting Factor 4, *PIF4,*. The high expression of sucrose-related genes depicts that they have significant effect on photomorphogenic responses. Generally, the high expression of carbohydrate-related genes was observed under the day-neutral treatment and down-regulated under the short-day treatment with higher expressions in WCC8 than in CC3. The latter (CC3) had some carbohydrate-related DEGs that were non-expressed, but which were expressed in SWCC8 (Figure 5). The dominant expression of carbohydrate-related genes shows that they may have important role in carotenogenesis under a long photoperiodic induction.

### 2.8. Identification of Differentially Expressed Genes Related to Carotenoid Biosynthesis

**Figure 6:**
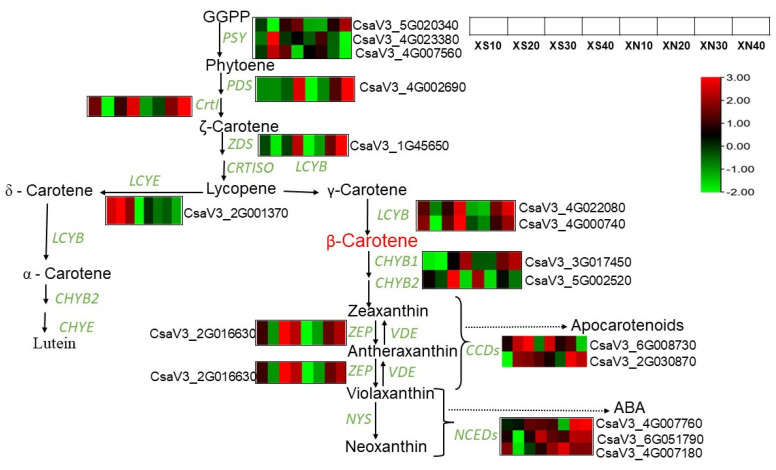
A schematic representation of the carotenoid biosynthetic pathway leading to carotenoid accumulation and degradation. The figure shows the expression pattern of each structural gene involved in carotenoid synthesis in XIS during fruit growth stages under photoperiod regimes. The color scale represents re-processed log10 (FPKM) using Pheatmap, representing the relative expression levels. The expression variance for each gene is indicated by colors ranging from low (**green**) to high (**red**). Geranylgeranyl diphosphate (GGPP); Phytoene synthase (*PSY*); Phytoene desaturase (*PDS*); Zeta-carotene Isomerase (*CrtI*); Zeta-carotene desaturase (*ZDS*); Carotene Isomerase (*CRTISO*); lycopene-β- cyclase (*LYB*); lycopene-ε- cyclase (*LYE*); carotenoid β-hydroxylase (*CHYB*); carotenoid-ε-hydroxylase (*CHYE*); Zeaxanthin epoxidase (*ZEP*); Violaxanthin de-epoxidase (*VDE*); Neoxanthin synthase (*NYS*); carotenoid cleavage dioxygenase *(CCD*); 9-cis-epoxycarotenoid dioxygenases (*NCEDs*); Abscisic acid (ABA).

We investigated the genes encoding enzymes involved in this pathway and their expression patterns (Figure 6, Datasheet S6). In general, the carotenoid biosynthetic pathway upstream genes, such as Phytoene synthase 1 (*PSY1*)*, ZDS* phytoene desaturase (*PDS*), 15-CRT*ISO* (*15-CRTISO*)and Zeta-desaturase (*ZDS)* showed a similar expression profile in XS and XN endocarp samples. Their expressions were down-regulated at earlier stages and then up-regulated at later stages with higher expression levels in XN than in XS. For example, *PSY1* at XN_40 DAP had an FPKM value of 490.75 compared to XS_40 DAP with an FPKM value of 365.195. The high β-carotene accumulation in XN could be linked to high expressions of these upstream genes. *LCYB* gene homologs (CSV3_4G022080 and CsaV3_4G000740) showed a similar expression pattern in both conditions in XIS, with low expression at early fruit growth stages and increased expression at 30 and 40 DAP. *LCD* expressed abundantly at earlier stages and then decreased at later stages, with up-regulation at XS10, XS20 and XS30 DAP and down-regulation at XS40DAP, and with downregulation throughout under XN at all time points DAP.

In this study, we observed a slight up-regulation of beta-carotene hydroxylase 1 *(CHYβ1*) (CsaV3_3G017450) at 40 DAP (FPKM = 1318.52) relative to XS at 40 DAPS (FPKM = 1129.618). This further emphasizes the putative role of *CHYβ1* under day-neutral conditions. Under XS, the level of Zeaxanthin epoxidase (*ZEP*) increased firstly at XS_10DAP, dropped at 20 DAPS, and then increased again at XS_30 and 40 DAP, and its level was slightly higher in XS (FPKM = 152.300) at 40 DAP than in XN (FPKM = 145.965), but with no significant difference (*p* = 0.05). For CS and CN, the *ZEP* expression increased further under CN (229.84) than in CS (18.96) at 40 DAPS, but in the flesh, its expression decreased firstly and then increased at mature fruit stages (Datasheet S6). The level of carotenoid cleavage dioxygenase (*CCD*) had a high expression at XN_30 and 40 and a low FPKM value was observed under XS. *CCD* unigenes (CsaV3_6G008730) increased at XS_20 and XS 30, and then declined at XS_40, with a decreasing trend also shown in XN over time. *CCD’s* (CsV3.2G030870) highest peak expression was realized at 40 DAP in the flesh of CC3 (Figure S9 and Datasheet S6). Carotenoid biosynthetic genes were more highly expressed under XIS than CC3, with high expression levels observed under XN. Carotenoid degradation genes are expressed more highly in CC3 than in XIS with higher FPKM values in CN. The analysis of the top ten DEGs in CS and CN revealed high expressions of carotenoid cleavage-related genes such as *WRKY* protein, phenylalanine ammonolyses, glycosyltransferase, lipoxygenase, and peroxidase, among others (Table S2).

### 2.9. Weighted Gene Co-Expression Networks Analysis (WGCNA)

**Figure 7:**
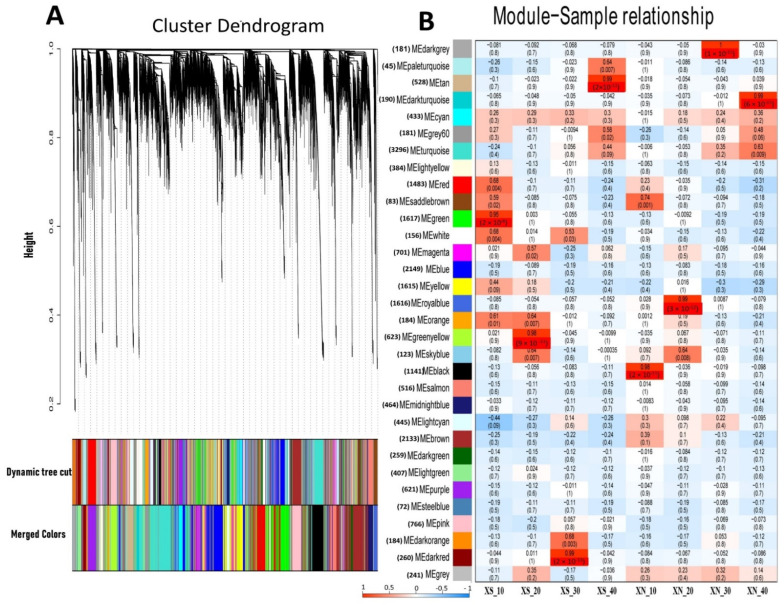
Establishment of WGCNA modules of the differentially expressed genes (DEGs) at the four stages of XIS (SWCC8)cucumber fruit development under short (XS) and day neutral (XN) photoperiod conditions. (**A**) Dendrogram of 21,742 DEGs obtained via hierarchical clustering of topological overlapping dissimilarity. (**B**) Relationships between module eigengenes (ME; rows) and sample at four stages (columns). The number in parenthesis against each eigengene is the number of DEGs. The color legend at the left corner underneath indicates the strength and direction of the correlation. Numbers within figure B are the partial Pearson correlations and the corresponding *p*-values. The upper value in each cell represents the correlation coefficient, and the lower value represents the *p*-value.

We used a co-expression network analysis employing WGCNA to gain an understanding of the regulation of the gene networks in the XIS cultivar under XS and XN conditions. Thirty-two unique modules (MEs) with various expression patterns were found using correlation patterns of the expressed genes (Figure 7, Datasheet S7). The gene counts for the standard modules ranged from 72 (MEsteal blue) to 3296 (MEturquoise), depending on their size. The clustering tree’s branches stand in for the individual modules. One gene is represented by each leaf. Every hue stands for a different module. By examining the correlation coefficients of the modules, we discovered that four modules had highly enriched genes (r > 90, P0.001) under XN, namely, MEblack (r = 0.98), MEroyalblue (r = 0.99), MEdark grey (r = 1), and MEdark turquoise (r = 0.99), at 10, 20, 30, and 40 DAP, respectively. On the other hand, four modules comprising MEgreen (r = 0.95), MEgreen_yellow (r = 0.98), MEdark_red (0.99), and MEtan (r = 0.99) were highly expressed at 10, 20, 30, and 40 DAP, respectively, under XS.

The gene functional categories that were enriched among significant modules were observed at different time points; β-carotene accumulation in the XIS cucumber (SWCC8) was observed during the mature fruit stage. Thus, in the later stages of fruit development, that is, 30 and 40 DAP, the dark-grey (XN_30DAP) modules were enriched for biological processes including “transport”, “response endogenous stimulus”, and “carbohydrate metabolic process”, and contained genes encoding starch and sucrose metabolism (Figure S10A, Datasheet S8). The dark turquoise module was enriched in genes associated with “carbohydrate metabolic processes”, “photosynthesis and light reactions”, “localization”, and “transport” (Figure S10B), and was also consistent with the GO enrichment of the DEGs (Figure 3A). The dark-red and tan modules (XS_30 and XS_40, respectively) were enriched in “cell wall organization”, “transport”, “response to stress”, “carbohydrate metabolic processes”, “catabolic processes”, “photosynthesis and light reactions”, and “signal transductions”. A two-component response regulator-like, *APRR5* gene which is involved in circadian rhythms, was among light signal transductions genes expressed in our co-expression analysis. The results indicate that carbohydrate metabolism genes and light signal transductions genes are strongly influenced by day length.. We identified the top 20 hub genes in both the MEdark-turquoise and MEdarkgrey groups (Table S4). Among the hub genes, significant genes that could have a potential role in carotenoid biosynthesis include to be light signal transduction E3 ubiquitin-protein ligase and Photosystem II CP43 reaction center protein), carotenoid-related genes (9′,10′-carotenoid cleavage dioxygenase), transcription factors (MYB and NAC), and genes involved in carbohydrate metabolism (β-glucosidase). In addition, we observed other hub genes whose functions in carotenoid metabolism or light induction remain unknown.

### 2.10. Correlating Network Modules with β-Carotene Accumulation in XIS Cucumber

**Figure 8:**
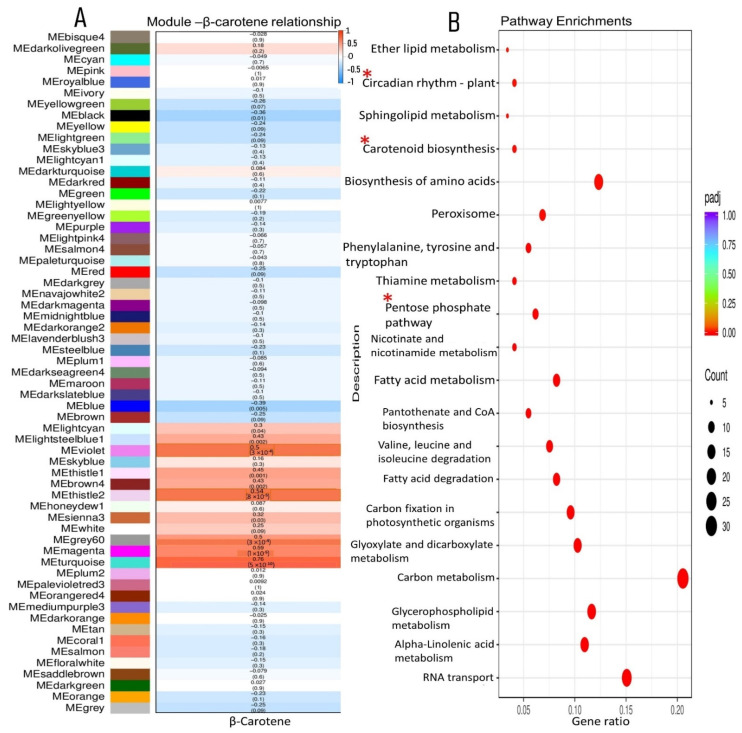
Co-expression networks and associated KEGG pathway enrichment of β-carotene and modules relationships. (**A**) Correlations between modules and β-carotene content; (**B**) Pathway enrichment of genes in the turquoise module positively correlated with β-carotene (R = 0.76). The red stars indicate the significant pathways of interest.

We focused on the identification of genes associated with carotenoid accumulation in the XIS cucumber and further used WGCNA to correlate the module eigengenes with the physiological variables (β-carotene). We found that the turquoise module had a positive correlation with beta-carotene content (r = 0.76) (Figure 8A). The KEGG analysis revealed that the enriched pathways in the turquoise module were mainly related to “carotenoid-related genes”,“circadian rhythm”, “tryptophan metabolism”, and the “Pentose phosphate pathway”, among others (Figure 8B). Seven circadian rhythm genes were observed these include Casein kinase II subunit alpha 1 (*CSNK2A1*), protein LHY-late isoform X2, E3, ubiquitin-protein ligase *COP1*, protein GIGANTEA-like, Two-component response regulators (APRR7) and Flavin-binding Kelch domain F-box Protein 1 (*FKF1*), *PIF3* isoform X1 (Datasheet S8). The carotenoid genes observed, namely, *ZDS*, *PDS*, *CHYβ-1*, *NCEDs*, and ABA8′hydroxylases, were equally co-expressed in the turquoise module (Datasheet S9). The co-expression of both carotenoid genes and circadian rhythm genes in the turquoise module suggests their interaction in regulating β-carotene accumulation in XIS cucumbers. The transcription factors MYBbHLH-WD40 (MBW complexes) are possibly the major determinant of carotenoid biosynthesis. Based on WGCNA, the turquoise module constituted 10 NAC TFs while MBW consisted of 7, 9, and 2 TFs, respectively (Figure S11). Other important TFs in the turquoise module include Bzip (2), SPX (3), AP2 (4), HD-ZIP (4), WRKY (7), and FARI (7).

### 2.11. Validation of Expression Profiles by qRT-PCR

**Figure 9:**
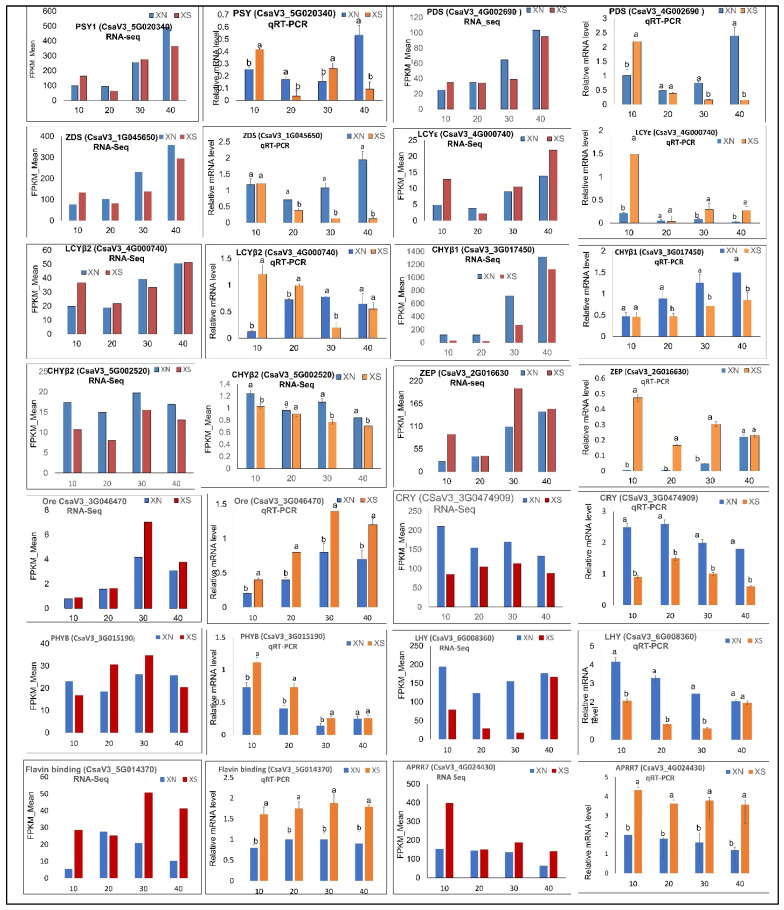
Validation of the expression of RNA-seq data by qRT-PCR using XIS (SWCC8) Cucumber cultivar. FPKM values were obtained via RNA-Seq and relative mRNA levels were obtained via qRT-PCR for putative carotenoid-related DEGs. Three technical replicates were performed for each biological replicate of each sample at 10, 20, 30, and 40 days after pollination. Error bars represent standard deviation. Different letters on bars at each sampling point indicate significant differences at *p* < 0.05. Phytoene synthase (*PSY*); Phytoene desaturase (*PDS*); Zeta-carotene desaturase (*ZDS*); lycopene-β- cyclase 2 (*LYB2*); lycopene-ε- cyclase (*LYE*); carotenoid β-hydroxylase (*CHYB1* and *2*); Zeaxanthin epoxidase (*ZEP*); orange gene *(Ore*); Cryptochrome (*Cry*); Phytochrome B (*PHY B*); late Elongated hypocotyl (*LHY*); Flavin binding Keltch F-box; Arabidopsis pseud-response regulator 7 (*APRR7*); Fragments Per Kilobase of transcript sequence per Million (FPKM).

We chose a total of 14 genes, namely, 9 genes linked to carotenoid production and 5 genes associated with light signal perception and transduction, and used qRT-PCR to analyze their expression patterns in order to further evaluate the validity and accuracy of the Illumina RNA-Seq study’s results (Figure 9). The expression profiles of each of the chosen genes matched those found in the RNA-Seq data. In light of the aforementioned facts, the validity of the RNA-Seq results was indicated.

## 3. Discussion

### 3.1. Photoperiod Regulation of Carotenoid Contents and Carotenoid Biosynthesis Pathway Genes in Cucumber Fruit Endocarp

Numerous factors influence the complex and tightly controlled biosynthesis of carotenoids in plants. Since carotenoids play significant role in shielding the photosynthetic machinery against excessive light, they are therefore, controlled by light components such as photoperiod [4,28,29]. In this study, we report the influence of photoperiod on carotenoid accumulation in photoperiod sensitive orange fleshed cucumber (XIS-SWWCC8) and compared to the non-photoperiod sensitive white fleshed type (CC3). In our study, the β-carotene accumulation in XIS (SWCC8) cucumbers was enhanced under day-neutral and reduced under the short-day treatments (Figure 1). This finding indicated that the endocarp’s coloration and the quantity of β-carotene in XIS fruits are light-dependent. Consistent with our result, day-neutral conditions or exposure to light have been reported to increase the accumulation of secondary metabolites such as anthocyanin in the leaves of malus crabapples [30], anthocyanin in litchi pericarp [31], carotenoids in mung bean sprouts [32], and carotenoids in the peel of mandarins and sweet oranges [33]. In addition, it has been observed that there are reduced carotenoid levels in the flesh of bagged peaches compared to those of non-bagged [34]. These results suggest that the photoperiod regulates metabolite accumulation and thus warrants molecular investigations.

Our results show that the day-neutral treatment of SWCC8 cucumber enhanced the expression of carotenoid-biosynthetic genes (*PSY1 PDS ZDS*, *LCYB1,2*, and *CHYβ1/2*) compared to a short-day treatment. A similar observation was recorded in CC3 but with lower expression levels than SWCC8. In addition, the higher expression of *LCY-*ε under the short-day treatment of SWCC8 further implies an increase in α-carotene and Lutein concentrations and a reduction in β-carotenes. However, *LCY-*ε was downregulated under XN, thus implying a reduced α-carotene accumulation in favor of β-carotene. The expression trend of the carotenoid pathway genes corresponds to the β-carotene levels in XS and XN and between genotypes. Orange gene (*Ore*) encoding a DnaJ cysteine-rich zinc finger protein was identified from an orange cauliflower mutant is known to have a role in initiating chromoplast biogenesis in plants... In this study, we observed a decreased transcript level of *Ore* under day neutral condition and an increased expression level under short day condition. Similarly, a lower transcript level of *ore* was observed under illumination compared to darkness in the roots of a carrot, an indication that light negatively regulates the expression of ore gene [5]. However, this trend is not in tandem with the levels of β-carotene in our current study, thus implying that factors other than *ore* play key roles in β-carotene accumulation in XIS cucumbers under long hours of light; further, it is in agreement with the earlier reports by Waters and others [35], who hypothesized that *ore* is not the only factor contributing to orange flesh in XIS cucumber. In the XIS cucumber, the quantitative trait locus analysis found the ore gene located in chromosome 3 [26] to be associated with β-carotene accumulation. Further, map-based cloning linked the loss of function of *CsaBCH1* to a non-synonymous mutation caused by the change from Alanine in a common cucumber to Asparagine in the XIS cucumber genotype [27]. In this study, we observed an up-regulation of *CsaBCH1* (*CHYβ1*) under XN compared to XS, which implies that longer light exposure stimulates its expression levels. The up-regulation or down-regulation of the carotenoid genes are coordinated by light receptors [34,36]; thus, we next investigated the expression of light photoreceptors in our transcriptome data.

### 3.2. Light Signaling and Related Transcription Associated with Carotenogenesis

For the precise perception of light conditions, plants require a variety of sensory photoreceptors [37]. There are four primary categories of photoreceptors, these include; PHYs *s* (*PHYA-PHY B, PHY C, PHY D* and *PHY E*), which absorb red and far-red light; *CRYs* (*CRY1, CRY2,* and *CRY3*); PHOTs (*PHOT1* and *PHOT2*), which detect UV-A and blue light; and UVRB, which detects UV-B [38,39]. In this study, 23 light receptors/circadian rhythm genes, were detected and these include *PHYs* (*PHY A and Phy B*), *CRY* 1 two *PKS* (*PKS1* and *PKS1*), chalcone synthase, Flavin binding, FL-T, two *GIGANTEA*, *LHY-like*, two *HY5s*, two CP-like (*CP21_like* and *CP7-like*), two APRR (*APRR5* and *APRR7*), and *COP1*.

The phytochromes PHYA and PHYB have been identified as being primarily responsible for controlling photomorphogenesis and the synthesis of carotenoids [5,9,40]. In our study, *PHYB* expression peaked at XS 20 and XS 30 before declining at XS 40, with expression generally being higher under XS than XN. PHYC was upregulated at XS_40 and XN_10. Phytochrome (*PHY*) and phytochrome-interacting factors’ (*PIF*) expression levels in the dark (short day) negatively regulate carotenoid structural genes *(PSY2*, *PDS*, *ZDS*, *LCYE*, and *CHYB1*). In agreement with our study, it has been reported that *PHYA*, *PHYB,* and *PARI* had high expression levels in underground, dark-grown carrot taproots with a reduction in their expression when exposed to light [5].

Many plant species possess multiple *CRY* genes such as *CRY1, CRY2*, and *CRY3* (*CRY-*DASH) and their functions have been elucidated in Arabidopsis [41]. In this work, we found that Putative CRY1 was more highly expressed in XIS during day-neutral conditions compared to short days. Taken together, we speculated that CRY1 participates in the endocarp cellular processes in cucumbers under long light conditions. The circadian rhythm gene, *LATE ELONGATED HYPOCOTYL* (*LHY*), located upstream in the circadian network negatively regulates the expression of *PRR7* and *PRR5.* Along with *LHY* and *CCA1*, PRR7 and PRR5 exhibit peaks in activity during a long day, which causes the morning cycle. COP1 is the primary regulator of photomorphogenesis in higher plants and functions as a RING-E3 ubiquitin ligase upstream of GIGANTEA (GI) and downstream of the photoreceptor [42]. *Cop1-like*, *GI*, and their homolog genes were found in our transcriptome data, and short days tended to increase their expression levels. Early flowering-3 (*ELF3*) is known to be rhythmically expressed on long days [43,44]. Similarly, we observed upregulation of *ELF* under day-neutral conditions than shorter day lengths; therefore, we speculated that flowering genes may be involved in the regulation of endocarp carotenogenesis in XIS cucumbers. In Arabidopsis, PIFs, a bZIP transcription factor, and Long Hypocotyl 5 (HY5) work antagonistically to mediate light-activated carotenogenesis. Phytochrome-interacting factors (PIFs), is a family of bHLH transcription factors and has been shown to directly link light signaling and transcriptional regulation of PSY gene to coordinately regulate both chlorophyll and carotenoid biosynthesis. In a dark condition or prolonged darkness, PIF reduces PSY expression through the direct interaction with G-box element of PSY promoter and subsequently reduces carotenoid biosynthesis and also suppresses photosynthesis activity [45]. As a result, it In both in vitro and in vivo experiments, Arabidopsis PIF1 directly binds to a G-box factor in the AtPSY promoter, repressing transcriptional expression of AtPSY and genes related to chloroplast and chlorophyll development [9]. Contrarily, HY5 activity is stimulated to enhance PSY transcription [46], thereby raising levels of carotenoids.

### 3.3. Genes Involved in Carbohydrate Metabolism Induced by Photoperiod in the Cucumber Fruit Endocarp

The gene ontology enrichment analysis of the DEGs at different time points revealed a high proportion of genes associated with transport and metabolism, particularly carbohydrate-related genes. Carotenoid biosynthesis requires a carbohydrate supply for assembling the carotenoid’s molecular backbone [47]. In our transcriptome data, we observed 92 starch and sucrose-related genes, with β-glucosidase and trehalose-6-phosphate phosphatase (*Tre6p*) accounting for 19.5 and 11.5%, respectively, and a higher expression was observed under XN. To balance the supply and demand for sucrose from developing sink organs, *Tre6P* regulates sucrose production [48]. *Tre6P* affects the *bZIP11* transcription factor, at least partially mediating changes in gene expression. Trehalose 6-phosphate and MYB27 have been found to reduce the accumulation of carbohydrates and anthocyanins in red-fleshed fruit [49]. Sucrose-phosphate synthase (*SPS*), a crucial enzyme in this pathway that is regulated by a number of effectors, might have its in vivo activity enhanced, increasing the amount of glucose that is synthesized. The data support the idea that the duration of the day affects the distribution of newly fixed carbon between soluble sugars and starch. The only organelles engaged in carotenoid biosynthesis are the plastids, which are also the locations of the metabolism of sugar and starchy carbohydrates. As a result, the buildup of carotenoids and the breakdown of starch may be influenced by a feedback mechanism for maintaining a supply of carotenoids for carotenogenesis [50].

### 3.4. Weighted Gene Co-Expression Network Analysis (WGCNA) Reveals Genes Involved in the Photoperiod-Mediated β-Carotene Accumulation

Here, we focused on the XIS (SWCC8) cucumber to identify significant modules regarding the photoperiod treatment using the WGCNA tool. The WGCNA assessment revealed huge gene clusters and regulatory networks linked to the same GO and KEGG pathways as those previously observed in Figure 3 and Figure 4, thus indicating the robustness of our WGCNA analysis. Among the DEGs in the biological pathway we identified six carbohydrate-related genes (*Chitinase, putative, glucan endo-1,3-beta-glucosidase 8-like, acidic endochitinase-like, Beta-amylase, Polygalacturonase, Glucan endo-1,3-beta-glucosidase,* and *Beta-glucosidase*) and one “DNA-directed RNA polymerase subunit beta” involved in photosynthesis and light reactions and, subsequently, involved in carbohydrate metabolism.

The top 20 hub genes in the dark turquoise module and dark grey modules corresponding to XN_40 and XN_30, respectively, identified genes involved in carotenoid metabolism and those involved in photomorphogenesis. These include E3-ubiquitin ligase, which is necessary for the light-induced degradation of PIF1 in Arabidopsis [51]; transcription factor families (MyB and NAC) involved in chlorophyll and carotenoid modulation [52,53]; plastid movement impaired 2, a new gene involved in normal blue light-induced chloroplast movements in Arabidopsis, which is thereby capable of modulating the photosynthetic potential of plant cells [54]; β-glucosidase, which is involved at the transcriptional level in fleshy fruit development [55]; and photosystem II reaction center polypeptide, a light-harvesting pigment-protein [56]. Further, the module–phenotype relationship revealed a possible co-expression between light signaling and carotenoid biosynthetic genes, thus suggesting a coordinated carotenoid biosynthesis and accumulation in the endocarp of XIS cucumbers (Figure 8B, Datasheet S9). The expression the known key transcription factor regulators that have been previously found to be involved in carotenoid biosynthesis coupled with photomorphogenic genes indicates the robustness of our co-expression analysis. Additional hub genes encoding transcription factors such as Zinc finger CCHC-type and RNA-binding motif-containing protein 1 were detected, whose function in regulating carotenoid synthesis needs further characterization. Previous studies have shown that MYB families as well as other TF families such as bZIP, NAC, and bHLH play significant roles in carotenoid biosynthesis [10]. A number of elements of fruit growth and development, including the synthesis of carotenoids, are regulated by transcription factor (TF) families, which play a vital role in gene expression [57,58]. We identified the TFs relating to carotenoid biosynthesis in the turquoise module co-expressing with β-carotene and the total carotenoid level. These results suggested their involvement in photoperiod-induced carotenoid biosynthesis. For example, NAC can activate the transcription of MYB [59] to promote carotenogenesis. It has been observed that MBW transcription factor complex expressions were mostly upregulated when litchi fruits were exposed to light and it was concluded that MBW could be involved in light-induced anthocyanin synthesis [30]. A basic Helix-Loop-Helix (BHLH) TF affects carotenoid accumulation in tomato fruit by acting indirectly in the carotenoid biosynthetic pathway [60].

**Figure 10:**
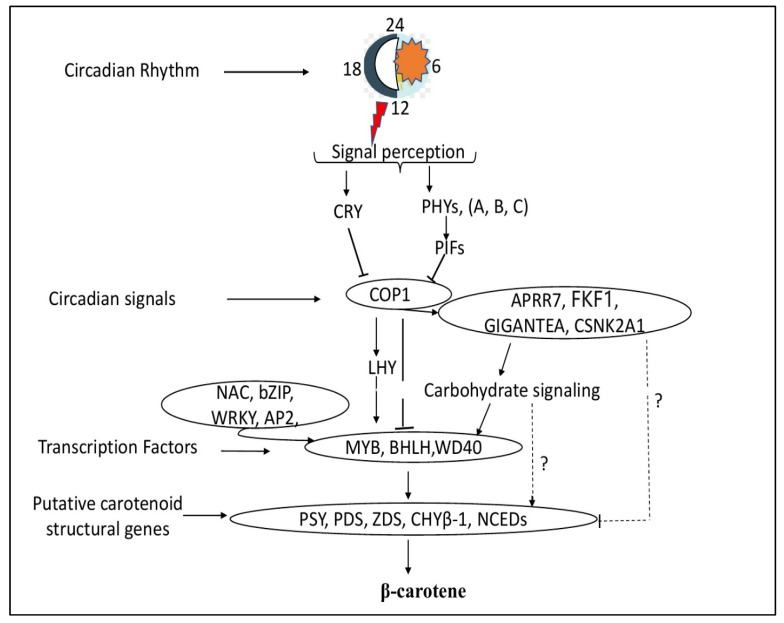
Schematic overview of the proposed model for photoperiod-induced β-carotene accumulation in the endocarp of XIS cucumber fruit. Arrows represent activation while T-bars represent repression. The gene names were the same as described in the main text. The question mark (?) denotes unknown molecular mechanisms involved between light signaling genes, carbohydrate-related genes, and putative carotenoid pathway genes.

Finally, based on our findings and previous studies involving model plants, we summarized a simplified model of photoperiod-regulated β-carotene biosynthesis in XIS cucumbers (Figure 10). Light signals are detected by CRYs and PHYs, which subsequently interact with *COP1*, a negative regulator that regulates the degradation of a number of light-response effectors, such as *LHY* and *HY5* [50,61], and other light-signaling genes. Alternately, *Cop1* can interact directly with MYB–BHLH–WD40 TF complexes to promote the transcription of genes involved in carotenoid structure synthesis, including *CsaBCH1*. Other circadian signaling genes that *COP1* interacts with include *APPR7* and the Flavin-binding Kelch F-box protein, which in turn interacts with genes that code for carotenoids. COP1 interacts with other circadian signaling genes such as *APPR7* and the flavin-binding Kelch F-box protein, which later interacts with carotenoid structural genes. Sugar-signaling genes promote the accumulation of the carbon backbone essential for carotenoid deposition. Sugars are also involved in this pathway; for example, the circadian oscillator gene GIGANTEA was reported to mediate a long-term response of the Arabidopsis circadian clock to sucrose [62]. In addition, sugar-responsive transcription factors such as *BZIP63* interact with carotenoid genes to induce carotenoid biosynthesis and accumulation [63]. The MBW complexes interact with other related TFs such as *NAC* and *bZIP* to induce the transcription of the carotenoid structural genes *PSY*, *PDS*, *ZDS*, *CHYβ-1*, and *NCEDs*. Therefore, the activation and expression of carotenoid pathway genes, either directly by photoperiod genes or through TFs, promote β-carotene accumulation in the fruit endocarp of XIS cucumbers.

## 4. Materials and Methods

### 4.1. Description of Plant Materials

Two inbred cucumber lines were used in this study, namely, the Chinese long inbred line (CC3) and a highly inbred XIS cucumber line (SWCC8). The SWCC8 line is a naturally occurring XIS cucumber that was discovered in Yunnan Province, China (21°09′ to 22°36′ N height, 99°58′ to 101°50′ E longitude, elevation 800–1200 m) and is a member of the Southwest China type [52]. SWCC8 has been classified as an SD plant; hence, it is photoperiod-sensitive [41,42]. On the other hand, the Chinese long inbred line (CC3) cultivar has long fruits with white flesh and is not photoperiod-sensitive.

### 4.2. Experimental Setup and Photoperiod Treatment

We conducted crop establishment and photoperiod treatments as outlined in our previous report [24] with minor alterations. The seeds of SWCC8 and CC3 were sown into a medium (Peat: vermiculite in a 3:1 ratio) and germinated in a growth chamber at 12 h/12 h (light/dark, ED) with a temperature of 27 °C/20 °C (day/night), 80% relative humidity, and 800 μmol·m^−2^·s^−1^ photon flux density. Uniformly sized seedlings with three fully unfolded true leaves were placed in the growth chamber for photoperiod treatments two weeks after germination. Artificial fluorescence tubes with white light in the SD (8 h/16 h, day/night) and equal-day (ED; 12 h/12 h, day/night) photoperiod configurations were utilized. The experiment used eight plants in pots filled with a 3:1 mixture of peat and vermiculite, with three replications for each treatment. Throughout the growth period, the plants were kept under these conditions. Regular management tasks such as nitrogen supplementation, pest and disease control, and irrigation were carried out. Fruit samples were collected at 10, 20, 30, and 40 days after pollination (DAP). Each sample was made up of three biological duplicates taken from three different plants. All of the seeds were discarded after the fruit endocarp was gently excised y using sterile surgical blades. After that, endocarp tissues were sliced into smaller pieces. For carotenoid and total RNA extraction, samples from three biological duplicates were divided into separate batches. Materials were immediately immersed in liquid nitrogen for a minute before being stored at −80 °C until use.

### 4.3. Carotenoid Extraction and High-Performance Liquid Chromatography (HPLC) Analysis

Carotenoids were extracted according to previously described method [64]. Briefly, fruit flesh portions (endocarp) were homogenized under liquid nitrogen, and 0.5 g samples of fruit flesh powder were placed into a 2 mL centrifuge tube. The samples were extracted with 500 μL of acetone-ethyl-acetate (EtOAc) (6:4 *v*/*v*) containing 1% butylated hydroxytoluene (BHT). The samples were centrifuged (Eppendorf centrifuge 5810R, New Brunswick, NJ, USA) at 4 °C for 10 min at 12,000 revolutions per minute (rpm). The upper ethyl-acetate phase (400 μL) was then transferred to new 2 mL microcentrifuge tubes and centrifuged for 5 min at the same rpm. The 300 uL of ethyl-acetate extracts was dried under a gentle stream of nitrogen, dissolved in 1 mL of acetone, and then stored at −20 °C until HPLC analysis. HPLC analysis of sample extract was carried out using a Waters Alliance 2695 system (Waters Corp., Milford, MA, USA). A 10 μL aliquot of each sample was injected into the HPLC column. Chromatography was carried out at 25 °C with an elution program described in [64]. Carotenoids were identified based on their retention times and spectroscopic characteristics compared with standards. Carotenoid concentrations were calculated by converting peak areas to molar concentrations by comparison with carotenoid standards of known concentrations determined by HPLC. The standards (β-carotene, violaxanthin, zeaxanthin, α-carotene, and lutein) were purchased from Sigma-Aldrich Co., Ltd., Shanghai, China.

### 4.4. Measurement of Carbohydrate Contents in the Endocarp of Cucumber Fruit

The samples utilized in this investigation were the same ones used in the carotenoid, RNA-seq, and qPCR array analyses, and they were all stored at −80 °C. Soluble sugars of glucose, fructose, and sucrose were determined using the approach in [65] with slight changes. Briefly, 200 mg of stored material was incubated for 15 min at 85 °C in 80 percent ethanol. After centrifugation at 12,000× *g* revolutions per minute (rpm) for 30 min, the supernatant was collected. The supernatant was stored at −20 °C, while the pellet went through a second extraction with 200 L of ddH_2_O. The supernatant was collected and dried at 60 °C under a vacuum, and then dissolved in 80 percent ethanol (*v*/*v*) and stored at −20 °C overnight. The suspension was centrifuged for 10 min at 4 °C at 20,000× *g*. To determine soluble sugars, we employed an HPLC-evaporative light-scattering detection (ELSD) approach. The HPLC-ELSD settings were optimized using a solvent ratio of 85 acetonitrile:15 water (*v*/*v*), a flow rate of 1 mL/min, and column and drift tube temperatures of 45 and 82 °C, respectively, and a nebulizer gas flow rate of 2 mL/min. Peaks were measured using HPLC-grade sugar calibration standards, such as glucose, fructose, and sucrose (Sigma–Aldrich, Shanghai, China). Starch was determined following the previously described methods [66]. The samples were extracted with 80% ethanol to remove free sugars. The sugar-free residues were hydrolyzed in pH.5.0 buffer using the thermostable amylase, Termamyl (Novo Industries Ltd., Copenhagen, Denmark), at 100 °C for 30 min. The released glucose oligomers were hydrolyzed to glucose with amyloglucosidase by overnight incubation. After being transferred to a volumetric flask, made up to volume, and filtered, the released glucose in the solution was determined by a glucose oxidase/peroxidase procedure. The starch content was calculated as glucose × 0.9.

### 4.5. Transcriptome Sequencing (RNA-Seq Analysis)

Sequencing was performed on Illumina HiSeqTM 2500 platform at Beijing Nuohezhiyuan Technology Co., Ltd., Beijing, China). The construction of the library and Illumina sequencing were performed according to a previously described method [67]. RNA integrity was assessed using the RNA Nano 6000 Assay Kit of the Bioanalyzer 2100 system (Agilent Technologies, Santa Clara, CA, USA). Total RNA was used as input material for the RNA sample preparations. Reference genome and gene model annotation files were downloaded from the cucumber genome website database directly (http://cucurbitgenomics.org/organism/20, accessed on 10 July 2021). Hisat2 V2.0.5 was used to build a reference genome index, and Hisat2 V2.0.5 was used to match paired-end clean reads to the reference genome. Feature Counts V1.5.0-P3 were used to count the read numbers and mapped to each gene. The expected number of Fragments Per Kilobase of transcript sequence per Million (FPKM) of each gene was then calculated based on the length of the gene and the read count was mapped to each gene.

### 4.6. Differential Expression Analysis, GO, and KEGG Enrichment Analysis

Differential expression analysis was performed on two conditions/groups (three biological replicates per condition) using the DESeq2 R program (1.20.0). DESeq2 provides statistical routines for evaluating the differential expression in digital gene expression data using a model based on the negative binomial distribution. The false discovery rate was regulated by adjusting the *p*-values using Hochberg’s and Benjamini’s method [68]. Before differential gene expression analysis, read counts for each sequenced library were modified by the edgeR software package by one scaling-normalized factor for genes with an adjusted *p*-value (For edgeR without biological replicates). The edgeR package was used to conduct a differential expression analysis of two conditions. The Benjamini and Hochberg method was used to modify the *p* values. The threshold for significant differential expression was set at a corrected *p*-value of 0.05 and 2 absolute fold change. The Cluster Profiler R program was used to perform enrichment analysis of differentially expressed genes, with gene length bias removed. Differentially expressed genes were judged highly enriched via GO terms with a corrected *p*-value of less than 0.05. Similarly, the program was used to examine the statistical enrichment of DEGs in the KEGG pathway.

### 4.7. Gene Co-Expression Network Analysis (WGCNA)

The WGCNA V1. 48 package was used to construct a gene co-expression network. In this study, the expression levels of 48 transcriptome samples (2 genotypes, 2 photoperiod conditions, 4 time points, and 3 replications) were used for WGCNA analysis. Co-expression network was constructed through the automatic network construction function block-wise modules. The network type was unsigned, and the correlation type was Pearson. In the modules, the exceptions were that the power was 9, the similarity threshold was 0.19, and the minimum number of genes was 20. To study the highly correlated modules, the Pearson correlation coefficient (r) between the sample matrix and the gene module was calculated and statistically tested. The larger the correlation coefficient, the higher the correlation between the module and the sample. We used Pearson’s correlation coefficient > 0.9 as a specific module–sample relationship and >0.7 as a module–trait relationship for further analysis.

### 4.8. Validation by Quantitative qRT-PCR Analysis

The fruit flesh samples similar to those used for RNA-seq were used in Real-time quantitative PCR to test the reliability of the RNA-seq data. Nine carotenoid-related DEGs and five light-signaling genes were selected. Primers were designed based on the http://cucurbitgenomics/20 database, accessed on 12 October 2021. The gene-specific primer pairs for qRT-PCR were designed from unique gene regions using Primer Premier 5.0 and listed in Table S3. Reverse transcription and fluorescence measurements were performed according to the requirements of the kit (Vazyme, Nanjing, China). Three technical replicates were performed for each biological replicate of each sample. The 2^−ΔΔCq^ method was used to calculate relative gene expression as described by Pfaffl [69]; the qRT-PCR data were analyzed with SPSS v.21.0 software for variance analysis, and the Waller–Duncan (W) method was used for comparison at the *p* < 0.05 level.

## 5. Conclusions

The biosynthesis and accumulation of plant metabolites are regulated by multiple environmental factors, and light signaling play an integral component Our study provides insights into the molecular mechanism involved in photoperiod-regulated β-carotene accumulation in orange-fleshed XIS (SWCC8) cucumbers. The results suggest that circadian signaling genes interact with the carbohydrate-related genes and other transcription factors to regulate carotenoid metabolism and accumulation. The study unveils potential genes that are applicable for future functional studies and molecular breeding of improved β-carotene rich cucumber varieties and other related crops.

## Data Availability

All relevant data analyzed during this study are included in this article and additional files. The RNA-seq data used in this study were deposited in the NCBI public database with the project accession number PRJNA782229 (https://www.ncbi.nlm.nih.gov/sra/PRJNA782229, accessed on 20 October 2021).

## Supplementary Materials

The following supporting information can be downloaded at: https://www.mdpi.com/article/10.3390/ijms232012650/s1.
