# Supplementary material for: Transcriptomic and Physiological Analyses Reveal Potential Genes Involved in Photoperiod-Regulated β-Carotene Accumulation Mechanisms in the Endocarp of Cucumber (Cucumis sativus L.) Fruit
Source: Int J Mol Sci. 2022 Oct 21;23(20):12650. doi: 10.3390/ijms232012650 (PMC9604348; doi:10.3390/ijms232012650)
Supplement: Supplementary file 1 [file ijms-23-12650-s001.zip › 1. Additional file 1_Tables.pdf]

Supplementary Materials

Supplementary Table S1: Statistics of Differentially Expressed Genes for all pairwise comparisons

		All-DEGs	Up-reg DEGs	Down-reg DEGs
Short Day (XS)	XS_40DAPvsXS_10DAP	12721	6346	6375
	XS_30DAPvsXS_10DAP	12174	6170	6004
	XS_20DAPvsXS_10DAP	11087	5646	5441
	XS_30DAPvsXS_20DAP	9594	4806	4788
	XS_40DAPvsXS_30DAP	9002	4462	4540
	**Total**	**54578**	**27430**	**27148**
		all	up	down
Day Neutral (XN)	XN_40DAPvsXN_10DAP	6585	2489	4096
	XN_30DAPvsXN_10DAP	2845	1037	1808
	XN_40DAPvsXN_30DAP	1943	723	1220
	XN_30DAPvsXN_20DAP	1586	903	683
	XN_20DAPvsXN_10DAP	1161	361	800
	**Total**	**12959**	**5152**	**7807**
XS VS XN		all	up	down
	XS_10DAPvsXN_10DAP	8135	3556	4579
	XS_40DAPvsXN_40DAP	5541	2882	2659
	XS_30DAPvsXN_30DAP	3933	1894	2039
	XS_20DAPvsXN_20DAP	2676	1031	1645
	**Total**	**20285**	**9363**	**10922**
		all	up	down
XS VS CS	XS_30DAPvsCS_30DAP	10862	5426	5436
	XS_10DAPvsCS_10DAP	10261	4848	5413
	XS_40DAPvsCS_40DAP	6259	2633	3626
	XS_20DAPvsCS_20DAP	5049	1932	3117
	**Total**	**32431**	**14839**	**17592**
		all	up	down
XN VS CS	XN_40DAPvsCN_40DAP	8767	4186	4581
	XN_30DAPvsCN_30DAP	1971	874	1097
	XN_20DAPvsCN_20DAP	1700	783	917
	XN_10DAPvsCN_10DAP	1385	785	600
	**Total**	**13823**	**6628**	**7195**
		all	up	down
Short Day (CS)	CS_30DAPvsCS_10DAP	7286	3559	3727
	CS_20DAPvsCS_10DAP	3107	1984	1123
	CS_30DAPvsCS_20DAP	2549	346	2203
	CS_40DAPvsCS_30DAP	2138	1779	359

	CS_40DAPvsCS_10DAP	1930	1212	718
	**Total**	**17010**	**8880**	**8130**
		all	up	down
Day Neutral (CN)	CN_40DAPvsCN_10DAP	5381	2233	3148
	CN_30DAPvsCN_20DAP	3807	1774	2033
	CN_40DAPvsCN_30DAP	3468	1653	1815
	CN_30DAPvsCN_10DAP	2906	1195	1711
	CN_20DAPvsCN_10DAP	399	209	190
	**Total**	**15961**	**7064**	**8897**
		all	up	down
CS VS CN	CS_30DAPvsCN_30DAP	7816	3627	4189
	CS_10DAPvsCN_10DAP	4588	1887	2701
	CS_40DAPvsCN_40DAP	3761	2135	1626
	CS_20DAPvsCN_20DAP	1381	772	609
	**Total**	**17546**	**8421**	**9125**

Table S2 : The top 5 DEGs with high fold change value (absolute value) and their annotation in the pairwise comparisons

**Gen ID**	**log_2FoldChange (XS vs XN_10 DAP)**	**Annotation**
CsaV3_5G034330	8.717070412	LOB domain-containing protein, putative
CsaV3_2G017980	7.909897915	terpene synthase 10-like isoform X1
CsaV3_5G024170	7.53420296	GAST1 protein homolog 5
CsaV3_4G001110	7.296776898	neuronal acetylcholine receptor subunit alpha-2-like
CsaV3_4G005340	7.253750836	Pectinesterase
CsaV3_5G023030	7.141934098	Zinc finger protein
	**XS_XN_20DAP**	
CsaV3_7G003200	8.012206344	O-methyltransferase
CsaV3_4G025340	7.134123668	Aldo-keto reductase family 4 member like
CsaV3_3G048700	7.122471646	Mediator of RNA polymerase II transcription subunit like
CsaV3_4G010440	6.638066969	Non-specific lipid-transfer protein like
CsaV3_2G027930	6.620749625	calcium-binding protein-like
	**XS_XN_30**	
CsaV3_4G010160	8.684881359	NADH-ubiquinone reductase complex 1 MLRQ
CsaV3_UNG139080	7.386646936	Diacylglycerol kinase family protein
CsaV3_UNG236470	7.091704679	Nicotinate-nucleotide pyrophosphorylase [carboxylating]
CsaV3_6G014370	6.939211794	Transcription factor
CsaV3_1G033960	6.835024934	p-hydroxybenzoic acid efflux pump subunit
	**XS_XN_40**	
CsaV3_1G022900	10.43824452	Trehalose 6-phosphate phosphatase

CsaV3_1G043750	9.82159882	aluminum-activated malate transporter 2-like
CsaV3_3G019090	8.197122636	nigrin b-like
CsaV3_6G000290	8.193268307	Cytochrome P450 family protein
CsaV3_7G018790	8.117529359	Protease inhibitor/seed storage/lipid transfer protein family protein
	**XS_20_vs_XS10DAP**	
CsaV3_1G028400	13.56626	**Flavin-containing monooxygenase**
CsaV3_6G047720	11.80599	Chitinase
CsaV3_5G038250	10.95801	DUF1997 family protein
CsaV3_6G046880	10.78118	**Cytochrome P450 protein**
CsaV3_5G003140	9.93433	Thaumatin-like protein
CsaV3_5G002490	9.884795	**Myb transcription factor**
	**XS_30_ vs_XS 10DAP**	
CsaV3_4G010160	12.70155	NADH-ubiquinone reductase complex 1 MLRQ subunit
CsaV3_6G000230	12.15289	Non-specific serine/threonine protein kinase
CsaV3_5G030890	11.34851	**Cytochrome P450**
CsaV3_7G008180	10.86775	Glycosyltransferase
CsaV3_3G014030	9.064308	**Sugar transporter ERD6-like**
	**XS_40_VS_XS10 DAP**	
CsaV3_2G004090	17.85852	Pathogenesis-related protein PR-4-like
CsaV3_7G000910	17.24173	protein NUCLEAR FUSION DEFECTIVE 4-like
CsaV3_2G035790	16.83134	benzyl alcohol O-benzoyltransferase
CsaV3_6G047720	16.70046	Chitinase
CsaV3_7G025280	16.06851	O-methyltransferase
CsaV3_1G028400	15.83893	Flavin-containing monooxygenase
	**XS_40_VS_XS30 DAP**	
CsaV3_3G014770	9.687555	Pollen Ole e 1 allergen/extensin
CsaV3_4G017770	7.961751	TATA-binding protein associated factor-like protein
CsaV3_3G004250	7.950353	nuclear transcription factor Y subunit B-9
CsaV3_1G030170	7.945444	Peroxidase
	**XN_20_VS_10DAP**	
CsaV3_1G031480	8.44305356	nicotianamine synthase-like
CsaV3_3G042480	8.08025781	**polygalacturonase QRT3-like**
CsaV3_4G000280	6.5465037	Glycyl-tRNA synthetase beta subunit
CsaV3_6G051190	6.28395822	DUF1264 domain-containing protein
CsaV3_7G030330	5.81060535	short-chain dehydrogenase reductase 3b-like
	**XN_30_VS_10DAP**	
CsaV3_7G027410	8.26139199	hyoscyamine 6-dioxygenase-like
CsaV3_5G000680	7.50836067	Bidirectional sugar transporter SWEET
CsaV3_1G043200	7.15431662	Cytochrome P450
CsaV3_2G004080	7.07481436	pathogenesis-related protein PR-4-like
CsaV3_4G000280	6.46865205	Glycyl-tRNA synthetase beta subunit

	**XN_30_VS_XN20DAP**	

CsaV3_5G002380	9.53858152	Endoglucanase
CsaV3_6G014100	8.87918387	kiwellin-like
CsaV3_3G000680	8.75002341	salicylate carboxy methyltransferase-like
CsaV3_3G044750	8.63126241	cytochrome P450 CYP82D47-like
CsaV3_4G003660	8.26440242	vinorine synthase-like
	XN_40_VS_XN10DAP	
CsaV3_5G032410	10.6819958	Pectate lyase
CsaV3_2G004070	9.60440314	pathogenesis-related protein PR-4-like
CsaV3_7G027410	8.10951072	hyoscyamine 6-dioxygenase-like
CsaV3_5G029570	8.0615112	WAT1-related protein
CsaV3_5G015610	7.99851178	cytochrome P450 CYP736A12
CsaV3_2G010730	6.92282594	protein PHYTOCHROME KINASE SUBSTRATE 1
	XN40_VS_XN30 DAP	
CsaV3_6G008100	6.50829635	proton pump-interactor 1-like
CsaV3_4G023650	6.26353379	Peroxidase
CsaV3_UNG045320	5.49799409	vesicle-fusing ATPase
CsaV3_3G048100	5.42818155	Rhamnogalacturonate lyase family protein
CsaV3_2G003870	5.33025949	VQ motif-containing protein
	XN_CN_10DAP	
CsaV3_2G001890	9.957394	Phosphotransferase
CsaV3_2G034850	9.012895	Unknown protein
CsaV3_3G008380	8.653469	Unknown protein
CsaV3_2G034840	8.575388	Unknown protein
CsaV3_4G034060	8.164979	Peptidase
	XN_CN_20DAP	
CsaV3_2G001890	9.871831	Phosphotransferase
CsaV3_4G032470	7.12886	Envelope-like protein
CsaV3_7G006630	7.038942	Glycosyltransferase
CsaV3_3G008200	7.02624	Vestitone reductase-like
CsaV3_2G027780	6.950865	Abscisic acid 8'-hydroxylase
	XN-CN_30DAP	
CsaV3_2G004070	9.925332	pathogenesis-related protein PR-4-like
CsaV3_4G032470	9.090304	Envelope-like protein
CsaV3_7G026270	8.096415	S-RNase
CsaV3_2G004080	7.965271	pathogenesis-related protein PR-4-like
CsaV3_1G009750	7.544343	MADS-box transcription factor
	XN_CN_40DAP	
CsaV3_7G008190	11.39395	Glycosyltransferase
CsaV3_2G035770	10.14854	benzyl alcohol O-benzoyltransferase
CsaV3_2G004070	9.286036	pathogenesis-related protein PR-4-like
CsaV3_3G040050	8.469035	Mediator of RNA polymerase II transcription subunit 15a-like protein
	**XS_10 VS_CS_10**	
CsaV3_1G008950	9.870239	Cytochrome P450

CsaV3_4G010130	9.761304	Alpha-1,4-glucan-protein synthase [UDP-forming] 1
CsaV3_2G008060	9.628639	Protein DETOXIFICATION
CsaV3_3G005390	9.432539	Polyubiquitin
CsaV3_1G030190	8.91818	SPX domain-containing protein
	**XS_20 CS_20DAP**	
CsaV3_1G031480	11.70235	nicotianamine synthase-like
CsaV3_4G010130	11.31719	Alpha-1,4-glucan-protein synthase [UDP-forming] 1
CsaV3_7G025710	10.44862	Unknown protein
CsaV3_3G001850	10.33202	Seed maturation protein
	**XS_30 CS_30DAP**	
CsaV3_1G031480	14.19585	nicotianamine synthase-like
CsaV3_1G010260	11.81587	Ankyrin repeat-containing protein
CsaV3_1G005600	10.98047	Chlorophyll a-b binding protein, chloroplastic
CsaV3_4G037920	10.94629	At4g19430
CsaV3_4G010130	10.65566	Alpha-1,4-glucan-protein synthase [UDP-forming] 1
CsaV3_4G010160	10.39904	NADH-ubiquinone reductase complex 1 MLRQ subunit
	**XS_40 CS40DAP**	
CsaV3_2G035790	12.8891	benzyl alcohol O-benzoyltransferase
CsaV3_5G014070	12.8694	Oxidoreductase, short chain dehydrogenase/reductase family
CsaV3_1G030190	11.8613	SPX domain-containing protein
CsaV3_2G004090	11.4109	pathogenesis-related protein PR-4-like
CsaV3_1G030210	10.9208	Cytochrome P450 family protein
	**CS_20VS_10DAP**	
CsaV3_3G042160	9.112987	WAT1-related protein
CsaV3_5G011080	8.936047	WRKY protein
CsaV3_7G002980	8.78654	Pathogen-induced protein CuPi1
CsaV3_1G044780	8.304662	Glucan endo-1,3-beta-glucosidase
CsaV3_5G000830	8.087867	Acidic endochitinase
CsaV3_5G037570	7.957281	metalloendoproteinase 2-MMP-like
	**CS_30_vs_CS10DAP**	
CsaV3_6G019010	9.040795	**Peroxidase**
CsaV3_6G039720	8.964182	**Phenylalanine ammonia-lyase**
CsaV3_3G036250	7.975705	thaumatin-like protein 1b
CsaV3_3G044690	7.376858	Cytochrome P450
CsaV3_3G014160	7.200929	CASP-like protein
CsaV3_2G004090	7.148448	pathogenesis-related protein PR-4-like
	**CS_30 VS_CS_20DAP**	
CsaV3_7G029080	6.990286	**desiccation-related protein PCC13-62-like**
CsaV3_2G031980	6.532487	EG45-like domain containing protein
CsaV3_6G052960	5.312135	alpha 1,4-glycosyltransferase family protein
CsaV3_UNG045320	5.025615	vesicle-fusing ATPase
CsaV3_1G043590	4.808247	Myb/SANT-like DNA-binding domain protein
	**CS40_VS_ CS10DAP**	
CsaV3_3G042160	8.29772	WAT1-related protein

CsaV3_5G011080	8.07481	WRKY protein
CsaV3_7G030280	7.48475	Protein nim1-interacting 1
CsaV3_6G039720	7.36969	**Phenylalanine ammonia-lyase**
CsaV3_5G001480	7.27209	Protein DMR6-LIKE OXYGENASE 2-like
CsaV3_6G042280	7.17608	WRKY family transcription factor
	CS_40 VS CS_30DAP	
CsaV3_1G005600	7.84857	Chlorophyll a-b binding protein, chloroplastic
CsaV3_1G009380	7.05494	vinorine synthase-like
CsaV3_1G038410	7.03947	F-box protein like
CsaV3_6G045990	6.98145	UDP-glycosyltransferase 72E1
CsaV3_3G039970	6.93256	WAT1-related protein
CsaV3_2G035510	6.90032	Glycosyltransferase
	**CN_20VS-CN 10DAP**	
CsaV3_4G010440	6.312347	Non-specific lipid-transfer protein like
CsaV3_UNG071590	6.240674	Malic enzyme
CsaV3_5G033430	5.987218	Cytochrome P450
CsaV3_5G008230	5.836786	Glycerol-3-phosphate acyltransferase
CsaV3_2G008150	5.80238	(R)-mandelonitrile lyase 1-like
	CN_30_VS_CN10DAP	
CsaV3_2G028090	7.873468	Alpha/beta hydrolase-3
CsaV3_3G036260	7.77962	Thaumatin
CsaV3_3G021910	7.678506	cation/H (+) antiporter 4-like
CsaV3_2G030870	7.444523	**Carotenoid cleavage dioxygenase**
	CN_30VS_CN20DAP	
CsaV3_6G047050	7.760112	protein WUSCHEL
CsaV3_1G015130	7.043541	NAC domain containing protein
CsaV3_1G046290	6.952583	Protein EXORDIUM-like
CsaV3_3G039060	6.943206	Cytochrome P450, putative
CsaV3_6G014480	6.829094	Carboxypeptidase
CsaV3_2G030870	6.567486	**Carotenoid cleavage dioxygenase**
	**CN_40VS CN_10**	
CsaV3_2G023210	9.476417	**Lipoxygenase**
CsaV3_3G010420	8.499599	Pectinesterase inhibitor
CsaV3_3G026530	8.22522	Gibberellin 2-beta-dioxygenase 2
CsaV3_6G006140	8.160713	LOB domain-containing protein 19
	**CN_40VS CN_20**	
CsaV3_1G005600	7.848574	Chlorophyll a-b binding protein, chloroplastic
CsaV3_1G009380	7.054937	Vinorine synthase-like
CsaV3_1G038410	7.039469	F-box protein like
CsaV3_6G045990	6.981452	UDP-glycosyltransferase 72E1
CsaV3_2G035510	6.900316	Glycosyltransferase
CsaV3_6G003270	6.835252	Cellulase (glycosyl hydrolase family 5) protein

CsaV3_5G001480	6.789136	protein DMR6-LIKE OXYGENASE 2-like
CsaV3_2G013640	6.7556	chlorophyllase-1-like
CsaV3_2G009640	6.718194	ABC transporter B family protein

Supplementary Table S3: Primer sequences used for qRT-PCR

		Primer sequences				
Gene Name	Gene ID	Forward primer (5’->3’)	Reverse primer (5’->3’)	Full length sequence	Product Size (bp)	Tm (^{0}C) F/R
CsaPSY1	CsaV3_5G020340	TTCATCGTTAGTTGTTTCTCCC	ATTCCCCCACTTCTGTCTCT	1266	157	56.5/54.9
CsaPdsa	CsaV3_4G002690	GTTCAGCCGATTTGATTTCCC	CGACTTCTCATCCACTCTTGC	1731	195	61.1/57.2
CsaZDs	CsaV3_1G045650	CAATGGAGAGAATGGGAGTT	CAATGGAGAGAATGGGAGTT	1587	170	53.8/54.8
CsaLCY-ε	CsaV3_2G001370	ATTACGGAAACTGCCACT	ACCTCAACCTCCACACCA	1602	170	49.8/52.8
CsaLCYβ2	CsaV3_4G000740	CCCAACATTCCTCTACGCA	TCTCTTCACCTTTATCCCC	1416	148	56.5/51.2
CsaCHYβ1	CsaV3_3G017450	GAGTCGCATCATAAACCAA	CCATCCCAAAGACCGTAA	933	166	51.3/53.6
CsaCHYβ2	CsaV3_5G002520	AAGAAAGCAGAAAGAGAGGG	GGGAAGAGAAACGGTAATAA	867	100	53.2/51.7
CsaZEP	CSaV3_2G016630	ACCGTGTGTTTTTGGGAC	CAGAATCTTCATCAGTGGC	1998	198	53/50.2
CsaOre	CsaV3_6G040750	GCCTTATTGCCCCAACTCTG	ATGACACCTACCGCTCCACC	975	145	59.7/59.5
CsaCRY1	CSaV3_3G047490	GCCAATGAAGGAAACAAAG	ACAACCCAGGGGAAGAAT	1839	152	49.5/50.9
CsaPHYB	CsaV3_3G015190	CCTCTCCAGTTCGTGTAA	AAGCCTTGTTGAGTTTCG	3399	194	47.7/50.5
CsaLHY-Like	CsaV3_6G008360	GGACTGCTACCTTTGTGTG	TGTTGGGAACTTTCTGCTT	2202	128	50.4/52.8
CsaFlavin binding	CsaV3_5G014370	CTGTATGATTGAAGGGTC	ATGTTGGCTTGTCTGTTGT	1890	107	50.6/50.1
CsaAPRR7	CsaV3_4G024430	TGAACCATAAAACGAGGAAG	CTAAAAAATCAACAGCACCC	2385	105	53/52.8

Tables S4. Top 25 Hub Genes Identified in the dark turquoise Module

Gene IDs	KME	Genne Annotation
CsaV3_2G034330	81.20932	CYC02 protein-like isoform X1
CsaV3_1G044120	80.57315	Jasmonate induced protein
CsaV3_2G004190	80.57315	Calmodulin
CsaV3_3G004290	80.57315	Nuclear transcription factor Y subunit B
CsaV3_3G020200	80.57315	Core-2/I-branching beta-1,6-N-acetylglucosaminyltransferase family protein
CsaV3_3G048570	80.57315	carotenoid 9,10(9',10')-cleavage dioxygenase 1-like isoform X1
CsaV3_4G003990	80.57315	Aldo/keto reductase
CsaV3_4G008650	80.57315	Pol polyprotein
CsaV3_4G017690	80.57315	Retrovirus-related Pol polyprotein from transposon TNT 1-94
CsaV3_4G026450	80.57315	Myb family transcription factor
CsaV3_5G003480	80.57315	Subtilisin-like protease
CsaV3_5G004070	80.57315	14 kDa proline-rich protein DC2.15-like
CsaV3_5G005120	80.57315	LRR receptor-like kinase
CsaV3_5G013890	80.57315	Non-specific lipid-transfer protein
CsaV3_5G026590	80.57315	Cysteine/Histidine-rich C1 domain family protein
CsaV3_6G003610	80.57315	Phosphate transporter
CsaV3_6G037670	80.57315	Late embryogenesis abundant protein
CsaV3_7G007320	80.57315	E3 ubiquitin-protein ligase
CsaV3_7G032980	80.57315	Acyl-[acyl-carrier-protein] hydrolase
CsaV3_UNG086680	80.57315	Methyl-CpG-binding domain-containing protein
CsaV3_UNG185410	80.57315	Zinc finger CCHC-type and RNA-binding motif-containing protein 1
CsaV3_UNG187480	80.57315	tRNA (Guanine(10)-N2)-methyltransferase homolog
CsaV3_UNG230070	80.57315	Beta-glucosidase
