# Supplementary material for: Transcriptomic and Physiological Analyses Reveal Potential Genes Involved in Photoperiod-Regulated β-Carotene Accumulation Mechanisms in the Endocarp of Cucumber (Cucumis sativus L.) Fruit
Source: Int J Mol Sci. 2022 Oct 21;23(20):12650. doi: 10.3390/ijms232012650 (PMC9604348; doi:10.3390/ijms232012650)
Supplement: Supplementary file 1 [file ijms-23-12650-s001.zip › 2. Additional File 2_Figures.pdf]

Supplementary Material

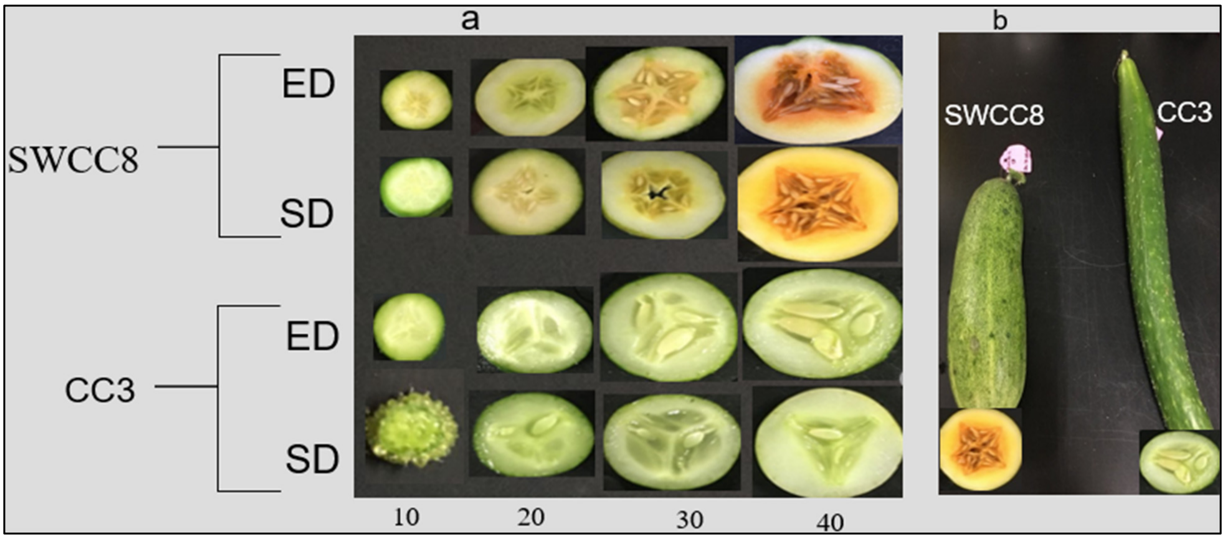

Figure S1. The phenotype of flesh color and morphology of cultivars and at different photoperiod regimes. (a)Flesh color changes in ‘SWCC8’ and ‘CC3’at four developmental time points (10, 20, 30 and 40 days after pollination). (b) Morphology of mature SWCC8 and CC3 fruits**.**

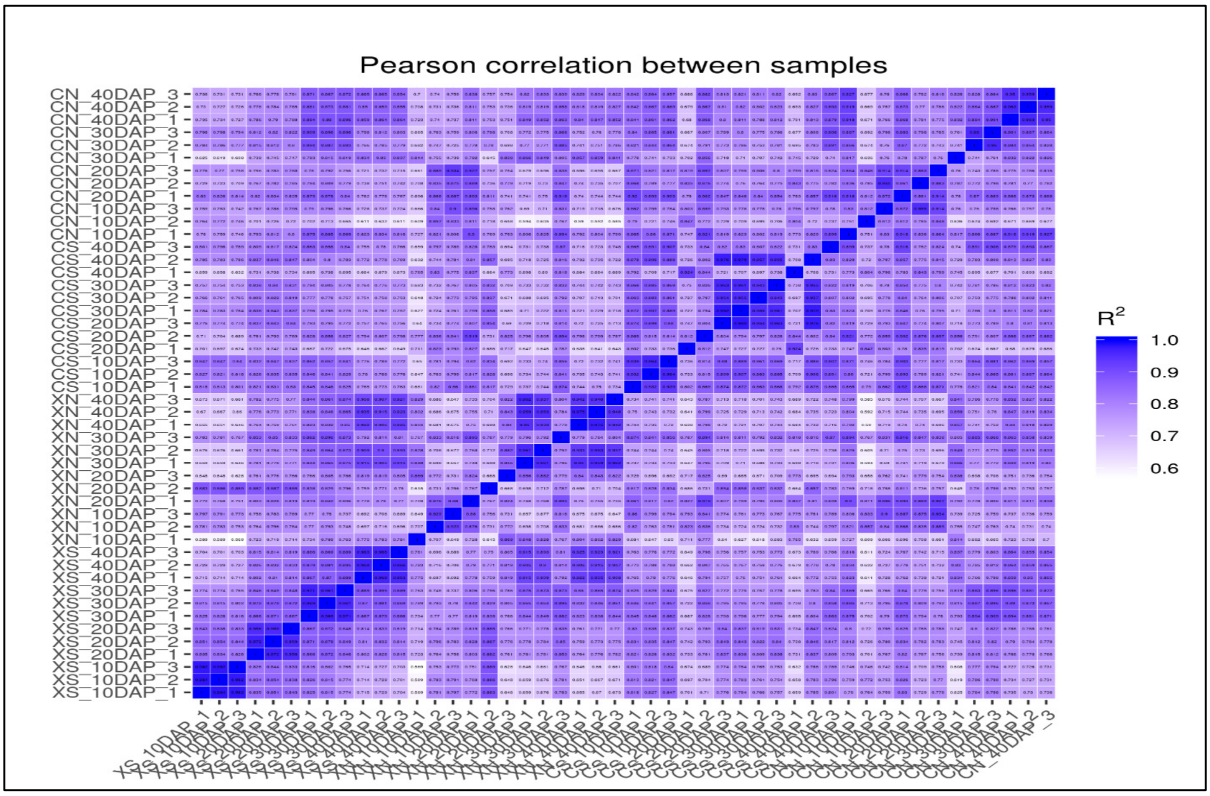

Figure S2: Pearson Correlation between samples. R^2 values between biological replicates were larger than those outside the biological replicates and the correlation coefficient in gene expression level from three biological replicates of each line were more than 0.84

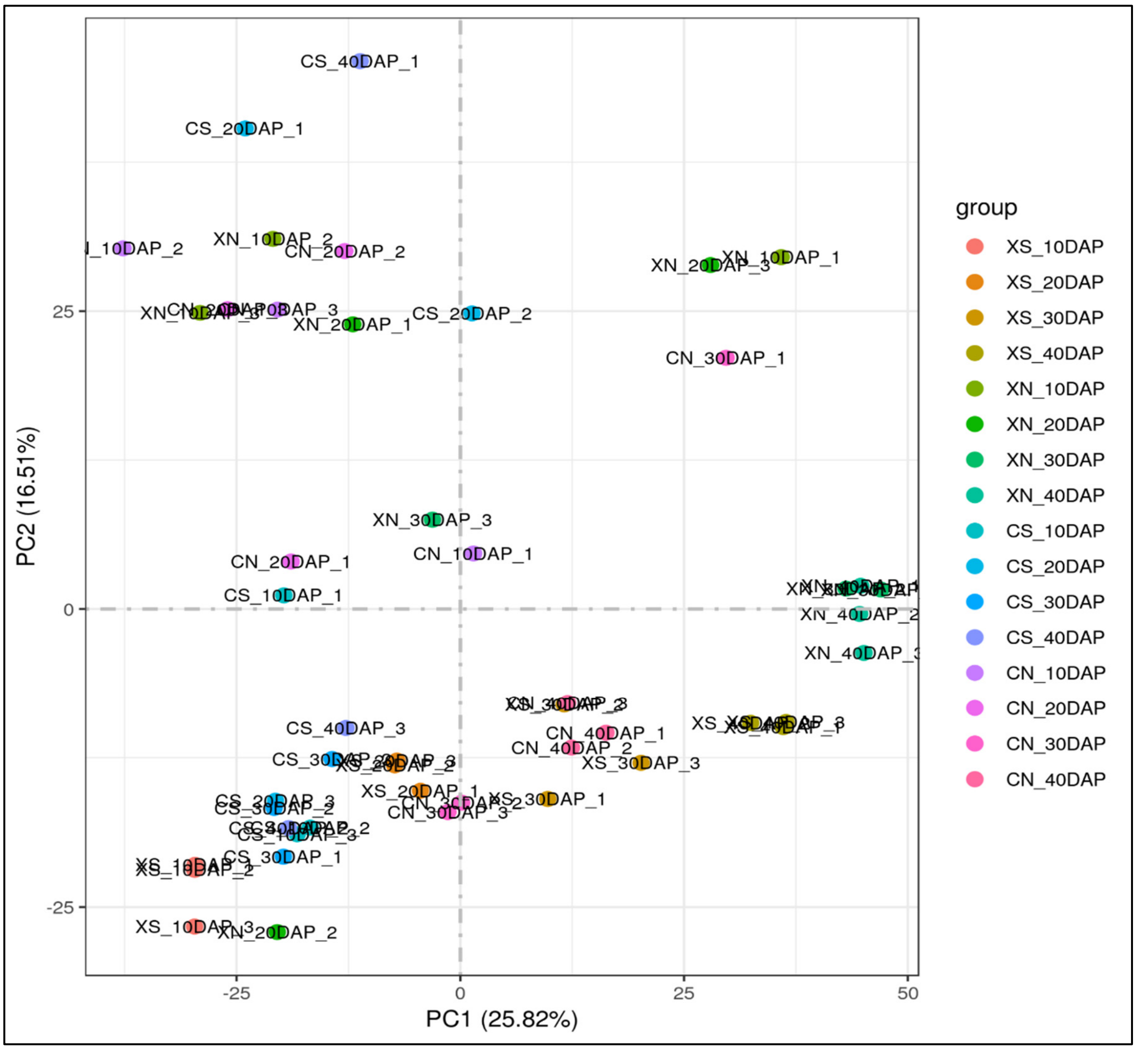

Figure S3: The principal component analysis (PCA) of the samples

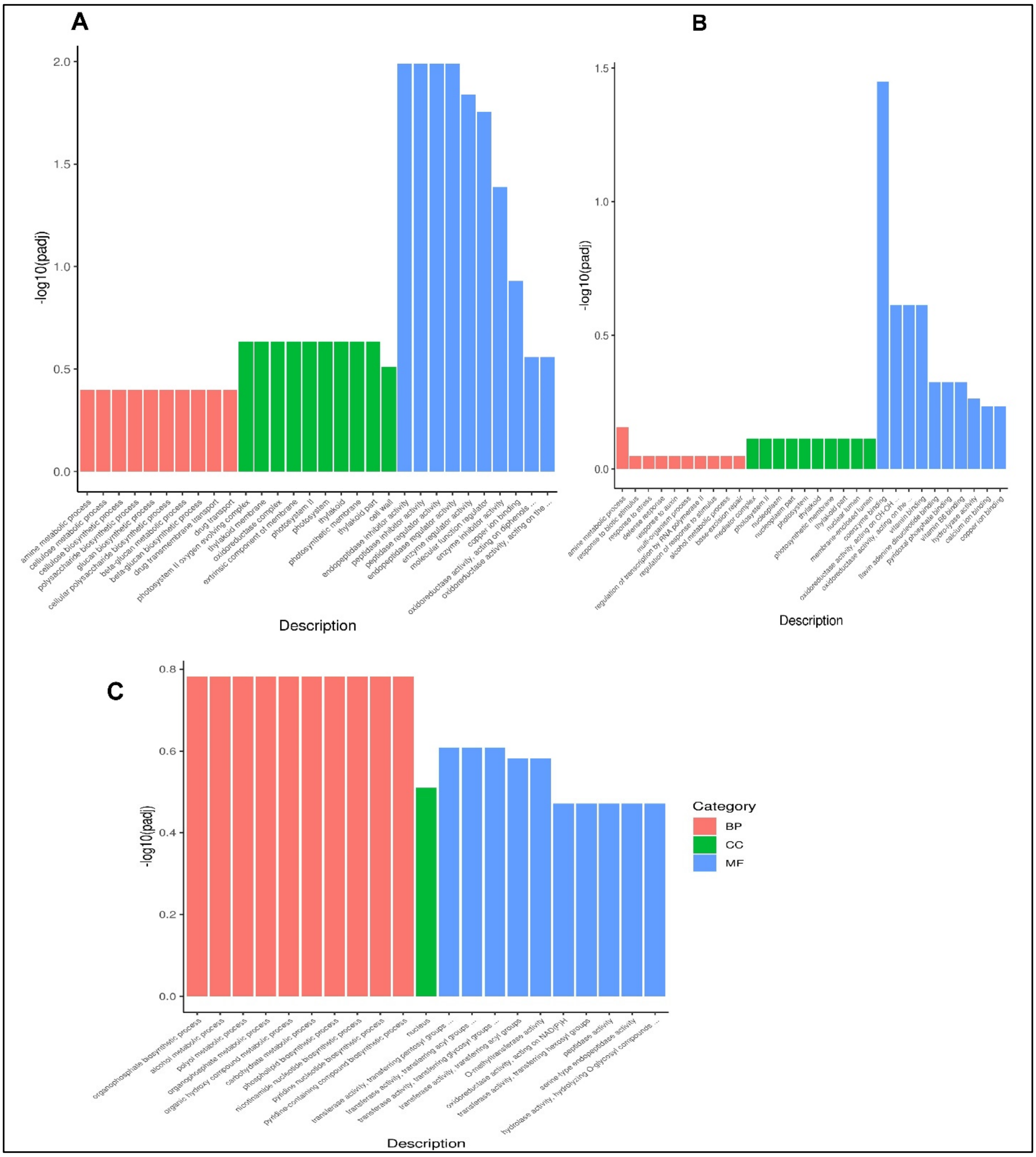

Figure S4. Gene ontology (GO) enrichment analysis in pairwise comparisons within and between CS and CN. (A) GO in the pairwise comparisons between CS_vs_CN. (B) Go terms of differentially expressed genes between CS pairwise timepoint comparisons. (C) Go terms of differentially expressed genes between CN pairwise timepoint comparisons.

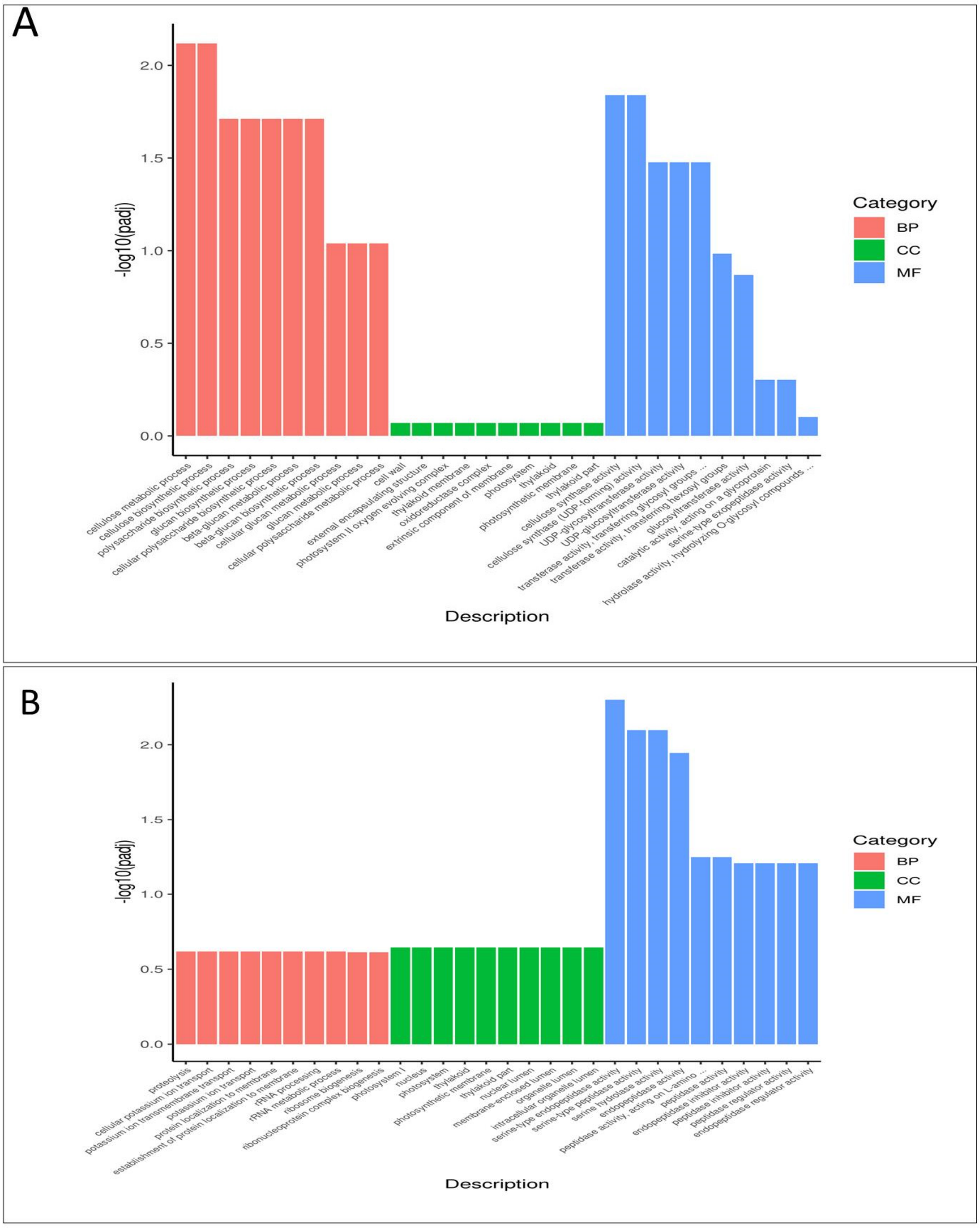

Figure S5. Commonly shared Gene ontology (GO) enrichment in pairwise comparisons between genotypes under similar photoperiodic effects of XS_vs_CS and XN_vs_CN. (A) GO in the pairwise comparisons between XS_vs_CS. (B) Go terms of differentially expressed genes between XN_vs_CN

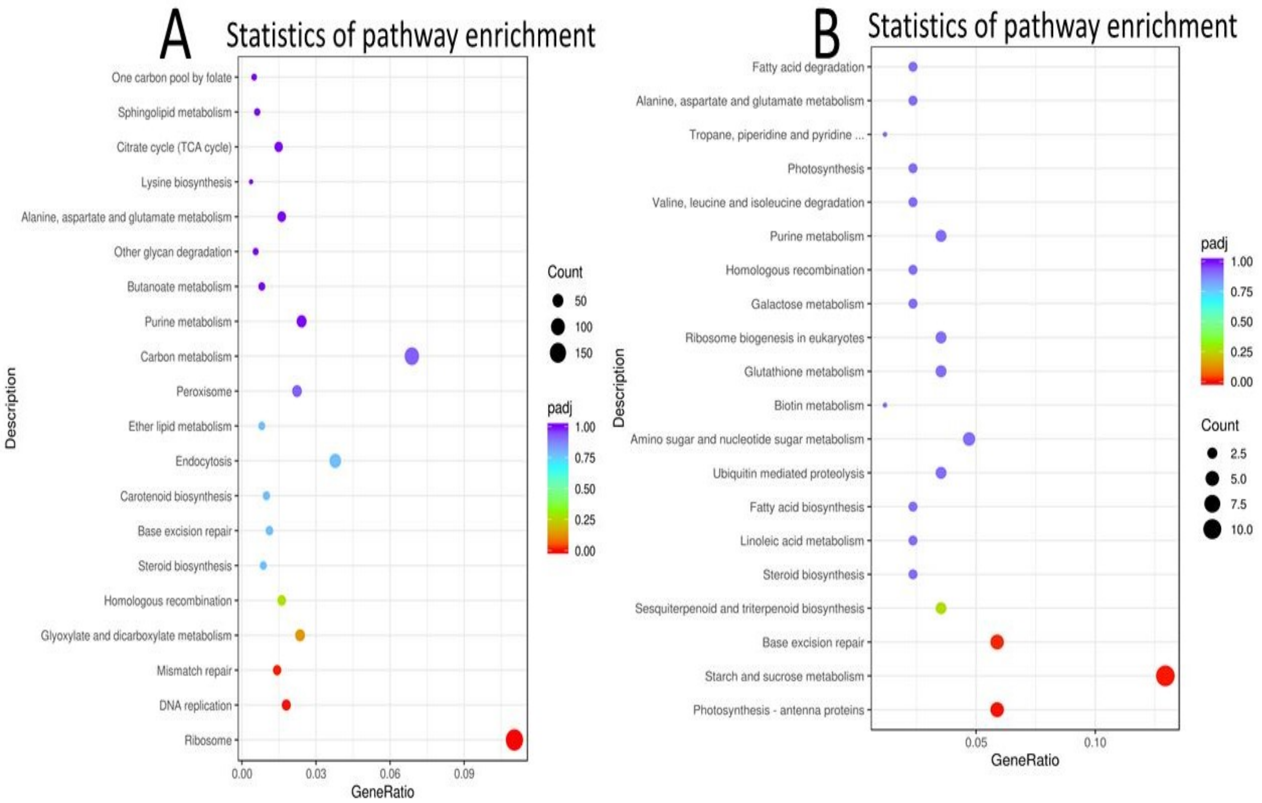

Figure S6. Significantly enriched KEGG pathways of DEGs in timepoint pairwise comparisons within XS and XN. (**A**) KEGG pathways of genes are commonly shared within timepoint pair-wise comparisons of XS treatments. (**B**) KEGG pathways of genes are commonly shared within timepoint pair-wise comparisons of XN treatments

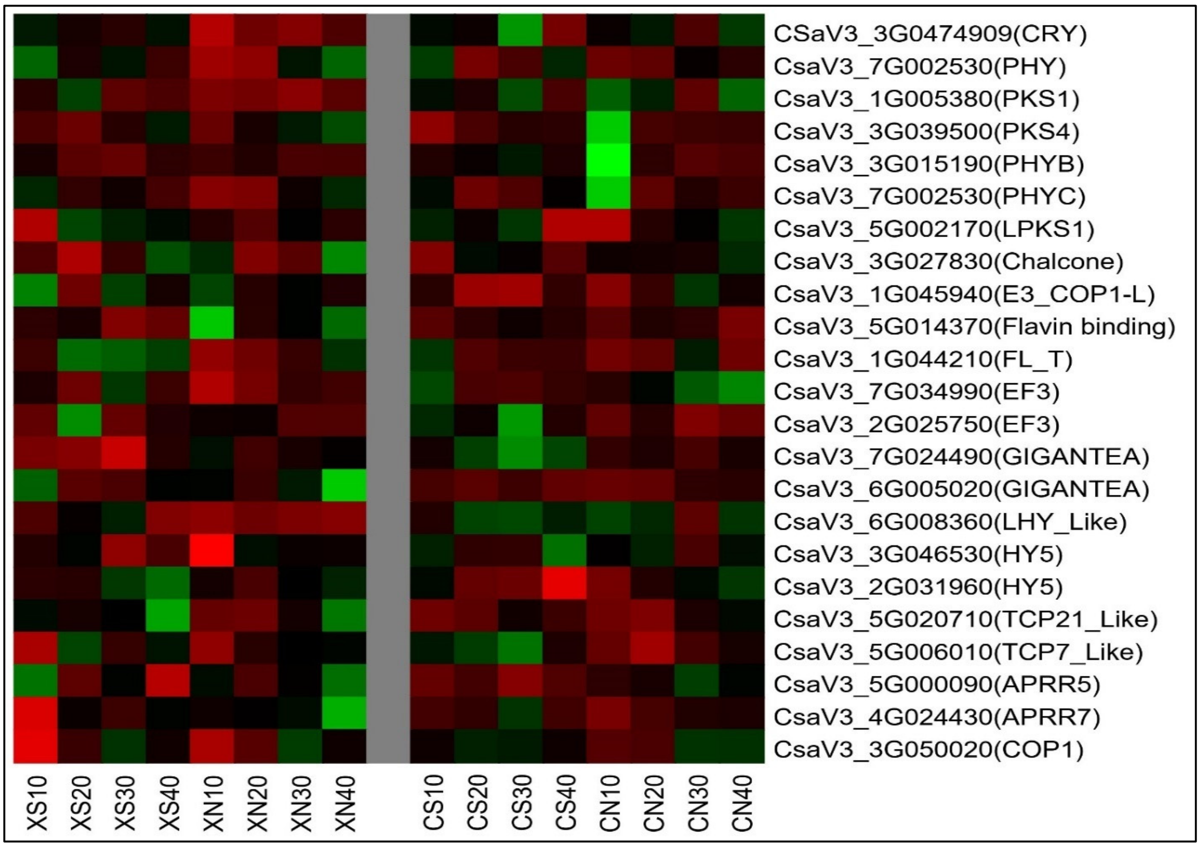

Figure S7. Expression profile of DEGs related to light signaling. Heatmap showing the expression of circadian and light signaling genes. The bars represent the expression scale for the DEGs (log_{10}FPKM) in each treatment.

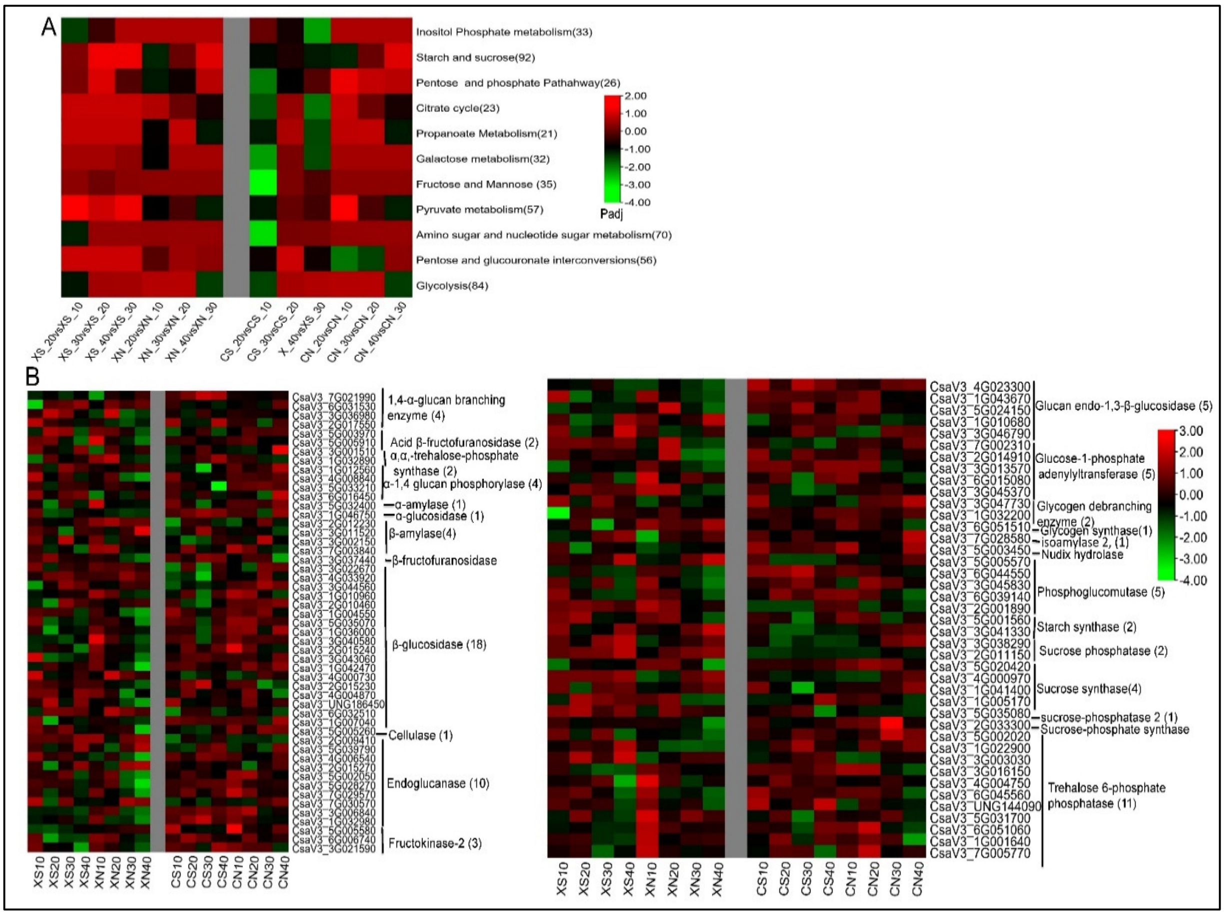

Figure S8. Expression profile of the genes involved in carbohydrate metabolism. **(A)** Heatmap showing the expression of major carbohydrates. **(B)** Heatmap showing expression levels of starch and sucrose-related genes. The number in parenthesis indicated the number of genes in each category. The bars represent the expression scale for the DEGs ($\log_{10}$ FPKM) in each treatment.

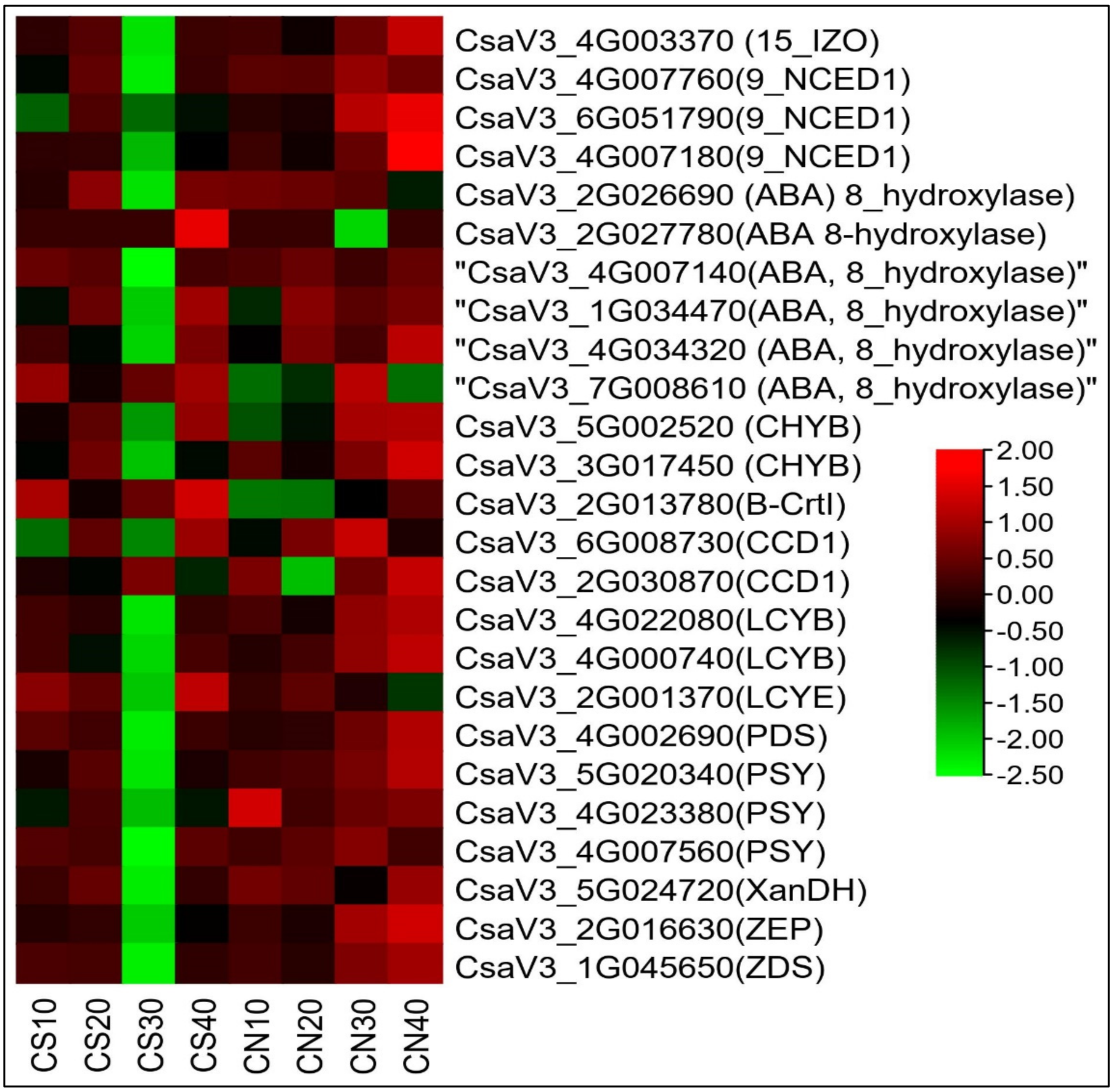

Figure S9. Heatmap of the expression pattern of each structural gene involved in carotenoid synthesis in CS and CN during fruit growth stages under photoperiod regimes. The color scale represents re-processed log10(FPKM) using Pheatmap, representing the relative expression level. The expression variance for each gene is indicated by colors ranging from low (green) to high (red).

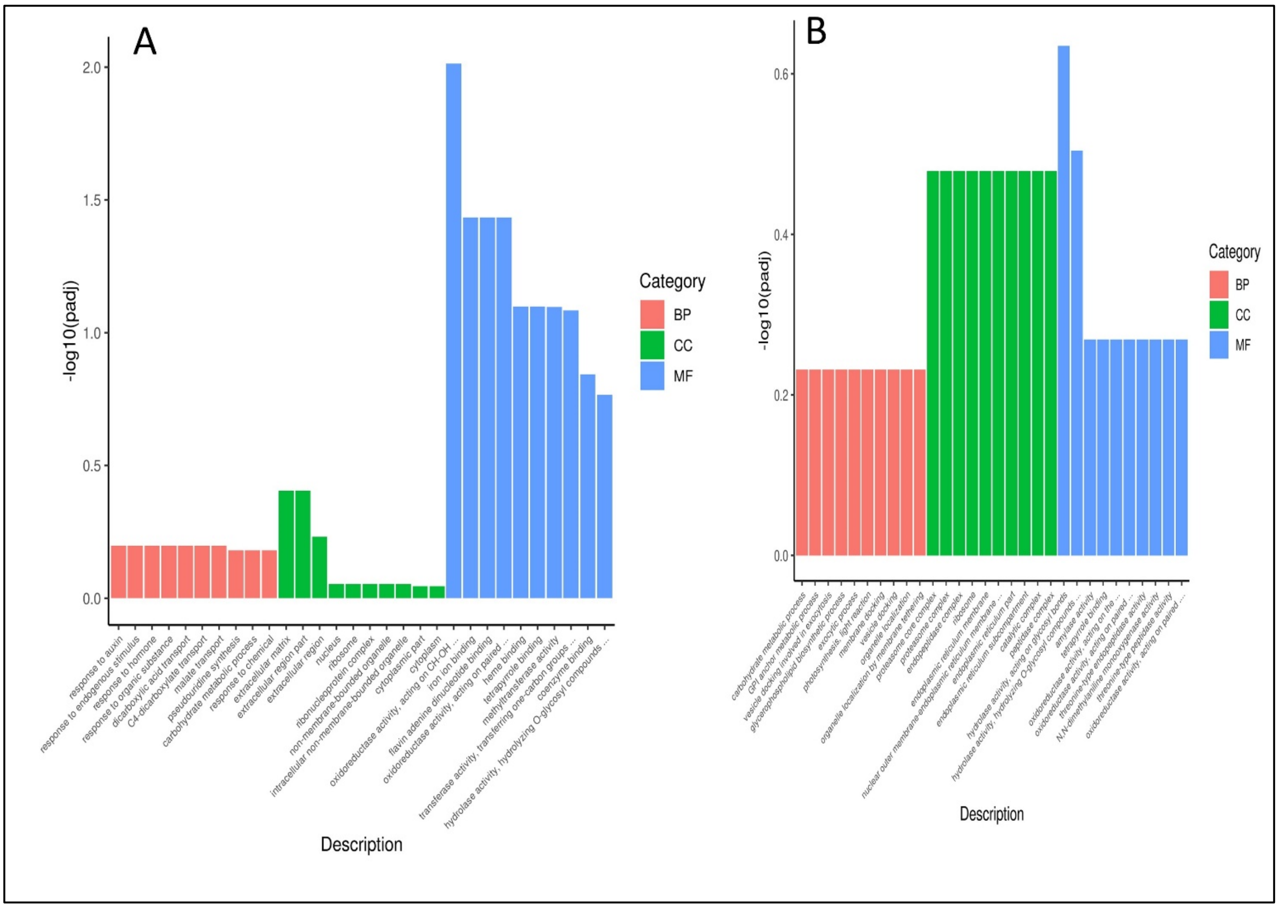

Figure S10. Module KEGG Enrichment in MEDark-grey module and turquoise module. (A) KEGG pathway of genes enriched in MEDark-grey module at XN_30DAP. (B) KEGG pathway of genes enriched in MEDark-turquoise module at XN_40DAP

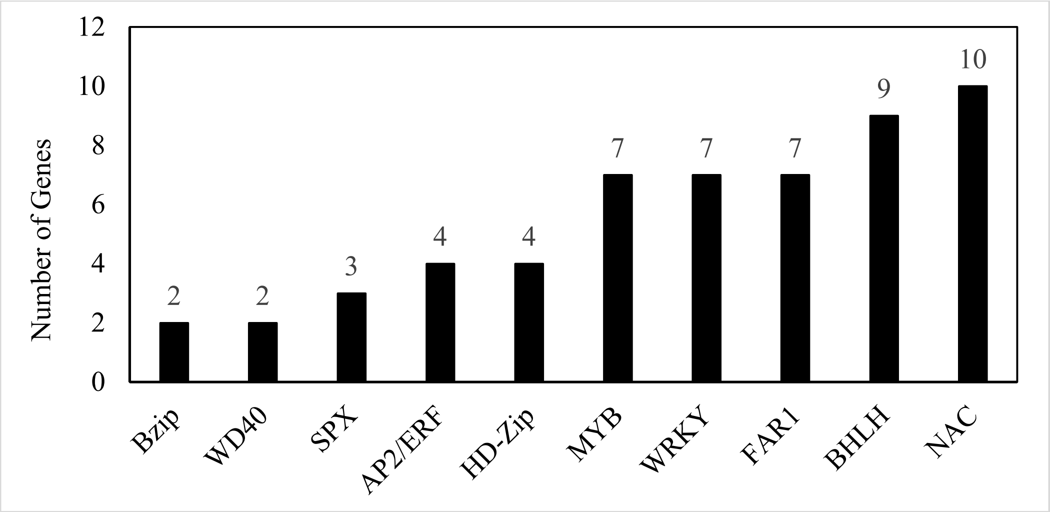

Figure S11. Distribution of transcription factors families related to carotenoid biosynthesis co-expressed in the turquoise module in the WGCNA of Module-trait relationship. Values on the bars are the number of genes in the TF family.
